# A role for the cortex in sleep-wake regulation

**DOI:** 10.1038/s41593-021-00894-6

**Published:** 2021-08-02

**Authors:** Lukas B. Krone, Tomoko Yamagata, Cristina Blanco-Duque, Mathilde C. C. Guillaumin, Martin C. Kahn, Vincent van der Vinne, Laura E. McKillop, Shu K. E. Tam, Stuart N. Peirson, Colin J. Akerman, Anna Hoerder-Suabedissen, Zoltán Molnár, Vladyslav V. Vyazovskiy

**Affiliations:** 1 Department of Physiology, Anatomy and Genetics, University of Oxford, UK; 2 Sleep and Circadian Neuroscience Institute, University of Oxford, UK; 3 Nuffield Department of Clinical Neurosciences, University of Oxford, UK; 4 The Picower Institute for Learning and Memory, Massachusetts Institute of Technology, MA, USA; 5 Institute for Neuroscience, Department of Health Sciences and Technology, ETH Zürich, CH; 6 Department of Biology, Williams College, MA, USA; 7 Department of Pharmacology, University of Oxford, UK

**Keywords:** sleep homeostasis, cortex, layer 5, EEG, slow-wave activity, sleep, SNAP25

## Abstract

Cortical and subcortical circuitry are thought to play distinct roles in the generation of sleep oscillations and global state control, respectively. Here we silenced a subset of neocortical layer 5 pyramidal and archicortical dentate gyrus granule cells in male mice by ablating SNAP25. This markedly increased wakefulness and reduced rebound of EEG slow-wave activity after sleep deprivation, suggesting a role for the cortex in both vigilance state control and sleep homeostasis.

The duration, timing and architecture of sleep are strictly regulated. Early studies based on neurological case reports, transections and electrical stimulation suggested that global state transitions are mediated via a distributed circuitry across the brainstem, the hypothalamus and the basal forebrain. More recent studies using selective targeting of specific neuronal populations based on their gene expression or connectivity patterns, highlighted that the sleep-wake promoting circuitry is highly complex, with distinct subcortical brain regions and neuronal subtypes responsible for specific aspects of wakefulness and sleep^1,2^. Although sleep-wake states are defined by the occurrence of neocortical and hippocampal oscillations, the possibility that neo- and archicortical neurons control vigilance states has been overlooked.

Cortical oscillations and neuronal firing patterns mirror sleep homeostasis^3,4^. Sleep homeostasis refers to the adjustment of the duration and intensity of sleep, to the duration of preceding wakefulness^4^. Electroencephalogram (EEG) slow-wave activity (SWA, EEG spectral power between 0.5-4 Hz) during NREM sleep represents a reliable marker of sleep-wake history^4^ and has been proposed to underlie many functions of sleep, such as cellular maintenance and synaptic plasticity. SWA can be regulated in a local, use-dependent manner^5,6^, in line with the view that sleep emerges within cortical networks driven by the local accumulation of metabolic products, such as adenosine^7^. However, slow waves also occur under anaesthesia, in isolated cortical slabs or even *ex vivo* ^8^. Therefore, the capacity to produce slow waves does not automatically imply a causative role for the cortex in physiological sleep or sleep homeostasis, either on a local or global level.

Here we test whether cortical structures have a function in regulating global sleep-wake dynamics. We focused on pyramidal neurons within layer 5 of the neocortex as well as archicortical dentate gyrus granule cells, two cell types involved in the generation of sleep oscillations. Layer 5 pyramidal neurons have been shown essential for the initiation and propagation of neocortical slow waves^9–11^, the main type of NREM sleep oscillations. Dentate gyrus granule cells contribute to the generation of hippocampal theta rhythm^12^, the defining oscillatory activity of REM sleep in rodents^13^. Yet neither of these two neo- and archicortical cell populations has so far been implicated in sleep-wake control.

Laminar local field potential (LFP) and multiunit activity (MUA) recordings were performed from the primary motor cortex of male adult wild type (C57BL/6) mice, concomitantly with EEG and electromyography (EMG) monitoring during 24 h undisturbed conditions (Fig. 1a-e). Consistent with the idea of an active role for layer 5 in generating slow waves^9,10,14^, we found that neurons in layer 5 tended to initiate spiking upon the onset of population ON periods (Fig. 1e, Extended Data Fig. 1). A leading role for layer 5 was indicated by a stronger initial surge of neuronal firing at OFF-ON transitions (Extended Data Fig. 1a), and a shorter latency to the first spike during ON periods, even when the total number of spikes in layer 5 was matched with layer 2/3 (Extended Data Fig. 1b).

To induce a cortex-wide reduction in the output from layer 5 pyramidal neurons, we used a transgenic mouse line, in which a subpopulation (~15-30 %) of pyramidal cells in layer 5 of the neocortex lack the key t-SNARE protein SNAP25 (Rbp4-Cre;Ai14;Snap25^fl/fl^)^15^. Rbp4-Cre is known as a pan layer 5 driver line (Fig. 1f, Supplementary Fig. 1)^16^, but also presents a strong Cre-expression in dentate gyrus granule cells (Extended Data Fig. 2). By contrast, Cre-expression outside of cortex is very sparse. Hypothalamic nuclei with established roles in sleep and circadian regulation show no or very few Cre-expressing cells and we found no overlap with orexin- or melanin-concentrating hormone-expressing cells in lateral hypothalamus (Extended Data Fig. 3-5). As has been shown previously, ablation of SNAP25 virtually abolishes calcium-evoked neurotransmitter release from neurons^15^, rendering the cells functionally silent. Importantly, normal brain development, including cortical layering and axonal path finding, has been shown in SNAP25-ablated mice^15^. We chose this conditional knockout mouse to probe a role for the cortex in sleep-wake regulation because in this model large and widely distributed populations of neo- and archicortical neurons involved in the generation of sleep oscillations and in the communication between cortex and subcortical structures are functionally silenced.

To investigate sleep architecture and electrophysiology in cortical SNAP25-ablated mice we performed chronic EEG, LFP, and MUA recordings as in wild type mice. Laminar MUA revealed a diminished surge of layer 5 firing at the onset of population ON periods (Fig. 1g). In addition, there were significant interaction effects between genotype and cortical layer for both slow wave amplitudes (*F*(1,8)=95.17, *p*<0.001) and SWA (*F*(1,8)=114.82, *p*<0.001). The amplitude of LFP slow waves in layer 5 was decreased (*t*(8)=3.70, *p*=0.006, *d*=2.34) and the levels of slow wave activity were reduced (*t*(8)=2.87, *p*=0.021, *d*=1.81, Fig. 1h,i). The opposite pattern was observed in layer 2/3, where a trend towards increased slow wave amplitudes (*t*(8)=-2.21, *p*=0.058, *d*=-1.40) and elevated slow wave activity (*t*(8)=3.07, *p*=0.15, *d*=-1.94) was observed (Fig. 1h,i). The differential regulation of layer 5 and 2/3 slow waves in cortical SNAP25-ablated mice results in a significantly reduced ratio of slow wave amplitudes and slow wave activity in cortical SNAP25-ablated mice (see insets on Fig. 1h,i). These layer-specific changes to intracortical dynamics are consistent with a reduction of local monosynaptic excitation between layer 5 pyramidal neurons^17^ and diminished disynaptic inhibition of layer 2/3 pyramidal neurons^18^. Laminar firing rates and the latency to the first spike in layer 5 were not significantly altered in cortical SNAP25-ablated mice in line with previous reports that abolition of evoked synaptic neurotransmitter release through SNAP25-ablation does not prevent neurons from depolarizing (Supplementary Fig. 2)^19^. The profound layer-specific changes in LFP slow wave activity during NREM sleep contrasted with a lack of major differences in EEG power spectra during NREM sleep or wakefulness (Fig. 1j and Supplementary Fig. 3). However, cortical SNAP25-ablated mice presented a leftward shift of the EEG theta-peak frequency during REM sleep (Extended Data Fig. 5). These layer- and sleep-state-specific findings underscore the importance of assessing sleep electrophysiology on the local and global levels^20^.

Beyond the local and global changes in sleep oscillations, we observed profound genotype differences in the daily sleep-wake profile (Fig. 2a). While control animals showed sleep architecture typical for wild type mice (Fig. 2b)^21^, cortical SNAP25-ablated animals presented unusually long wake bouts, that often lasted several hours (Fig. 2b,e). On average, conditional knockout mice spent 13.83±0.39 h awake per day, approximately three hours more than controls (10.57±0.42 h, *t*(13)=5.55, *p*<0.001, *d*=2.96), and the amount of sleep decreased proportionally (Fig. 2c). The differences between genotypes were more pronounced in the dark period (Fig. 2d and Extended Data Fig. 6), which is the mouse’s circadian active period but also the time of day when the homeostatic sleep drive typically builds up to high levels as a result of prolonged wakefulness^21^. This raises the question whether the increase in wakefulness is due to changes in the homeostatic or circadian process of sleep regulation.

To assess the build-up of the homeostatic sleep drive during spontaneous wakefulness, we compared the levels of EEG SWA during NREM sleep preceding and following individual wake episodes. As expected, a positive correlation was observed in both genotypes, whereby longer spontaneous wake episodes were followed by proportionally higher levels of SWA during NREM sleep. However, the increase of SWA relative to the duration of wake episodes was smaller in conditional knockout mice compared to controls (Fig. 2f, Supplementary Table 1), indicating that the relationship between sleep-wake history and the levels of SWA might be altered in cortical SNAP25-ablated animals.

An established approach to investigate the dynamics of sleep homeostasis is sleep deprivation (SD), which is most commonly performed starting at light onset, when mice in laboratory conditions usually sleep^3,21^. Typically, SD leads to a small increase in sleep amount, especially NREM sleep, and a strong increase in sleep intensity, reflected in SWA during NREM sleep^21,22^. However, we observed a striking difference in this homeostatic rebound between genotypes (Fig. 3a-c). Although many cortical SNAP25-ablated animals had already been spontaneously awake by the time sleep deprivation started (Supplementary Fig. 4a), they did not spend more time asleep after SD than controls when sleep deprivation was performed during the first half of the light period (Extended Data Fig. 7). When the time window of the sleep deprivation was shifted to the second half of the light period, the relative amount of sleep over the following 24 hours was reduced in cortical SNAP25-ablated mice compared to controls (Supplementary Fig. 4b). Moreover, conditional knockout mice presented a marked attenuation of the initial increase of EEG SWA during NREM sleep after sleep deprivation (relative SWA in cortical SNAP25-ablated: 136.77±3.98 %, controls: 180.57±5.13 %, *t*(11)=6.78, p<0.001, *d*=-3.87 Fig. 3b,c). Consistent with the notion of a frontal predominance of the homeostatic response to sleep deprivation^23^, the genotype difference in SWA rebound was observed in the frontal EEG and LFPs, but not in the occipital EEG derivation (Extended Data Fig. 8). We also observed that the increase in EEG theta-activity, a measure of ‘wake intensity’, was attenuated in conditional knockout mice during SD in both the frontal and occipital EEG derivation (Extended Data Fig. 9).

Since the circadian and the homeostatic process interact in the regulation of sleep and wakefulness, we next assessed whether the clock function was altered in conditional knockout mice, using a separate cohort of animals. A standard approach in circadian phenotyping consists in continuously monitoring locomotor activity using a passive infrared recording system under a 12:12 light:dark cycle followed by a release into constant darkness and exposure to a phase-advancing light pulse during the early subjective night (Circadian Time ~13.5) (Fig. 3d)^24^. We replicated the approximately three-hour difference in the amount of wakefulness in this new cohort of animals (cortical SNAP25-ablated: 11.79±0.29 h, controls: 9.10±0.51 h, *t*(15)=4.957, *p*<0.001, *d*=2.35) and observed that the sleep phenotype remained stable in constant darkness (Extended Data Fig. 10). Importantly, cortical SNAP25-ablated animals remained rhythmic in the absence of light (Fig. 3d). The free-running period was slightly shorter than 24 h and nearly identical in both genotypes (cortical SNAP25-ablated: 23.86±0.04 h, controls: 23.80±0.07 h, *t*(15)=0.833, *p*=0.42, *d*=0.39) and the Chi-Square periodogram analysis did not reveal any difference in circadian amplitude (Fig. 3e). Finally, irrespective of genotype, the light pulse evoked a consistent phase delay, which was comparable between genotypes (Fig. 3f). Taken together, our data demonstrate that cortical SNAP25 ablation leads to a diminished homeostatic sleep drive without affecting circadian regulation of sleep.

The regulation of sleep in mammals is only partially understood because it is unclear where and in what form the need to sleep is encoded, and how it is translated into an adequate compensatory response^25^. Our study reveals a novel role for the cortex in sleep-wake regulation. We show that cortical structures actively contribute to sleep homeostasis and the global control of vigilance states. This supports the hypothesis that brain structures fundamentally involved in sleep regulation extend far beyond the traditionally considered subcortical circuitry^2,7,26,27^. The next step will be to understand how neo- and archicortical neurons interact with established circuits of sleep-wake control. It was recently reported that axons of layer 5 pyramidal neurons in prefrontal cortex produce an axonal terminal field in the lateral hypothalamus^28^. Consistent with this observation, we found dense fine Cre-positive fibres surrounding cell bodies in the lateral hypothalamus (Extended Data Fig. 4). Long ranging projections from prefrontal cortex, which is known to be highly sensitive to homeostatic sleep pressure^23^ and a hub for the generation of slow waves during NREM sleep^29^, might represent a direct pathway through which neocortex could modulate the vigilance states control by the lateral hypothalamus. However, layer 5 pyramidal neurons also project to thalamic nuclei, and could influence sleep regulation through corticothalamic loops^30,31^. Given that layer 5 pyramidal neurons have a wide range of efferent connections to target structures involved in sleep-wake control, a systematic and unbiased dissection of the relevant circuits is warranted. Furthermore, a potential involvement of other neocortical cell types and of archicortical dentate gyrus cells of the hippocampus should be considered. Given the critical place of the hippocampus in brain-wide circuitry involved in memory and temporal processing, one could speculate that this structure may have a so far unrecognised role in encoding time spent awake or asleep. Targeted manipulations of the hippocampus and its neocortical projections could shed further light on the relationship between REM and NREM oscillations and their role in global sleep-wake regulation and function^32^.

Our results support the possibility that cortical structures generate sleep drive locally, in an activity-dependent fashion^7^, and raise the question of which mechanisms cortex could use to generate, sense and/or integrate signals of sleep need. Extracellular signals may be found in molecular regulators of inflammation and plasticity^7^, or adenosine levels regulated through neuro-glial interactions. Intracellular processes reflecting wake-dependent increases in sleep need, conserved both in mammalian and non-mammalian species, may represent changes in the synaptic phosphoproteome, endoplasmic reticulum stress, or redox homeostasis. Arguably, such local signals must be integrated to elicit a global homeostatic response^33^, reflected in an occurrence of intense sleep, characterised by elevated cortical SWA and increased sleep propensity. We propose that the wide connectivity of layer 5 pyramidal neurons to other parts of cortex, thalamus, and sleep-wake regulating nuclei in hypothalamus^28^ and brainstem^16^, places this neuronal population in an ideal position not only to generate SWA, but also to sense and integrate the signals related to sleep need, and ultimately broadcast the information to the subcortical circuitry responsible for sleep-wake switching^1^.

## Methods

### Animals

Rbp4-Cre;Ai14;Snap25^fl/fl^ is a triple transgenic mouse line, which was designed as a model for functional silencing of cortical layer 5 pyramidal and dentate gyrus granule cells. Snap25^fl/fl^ is a transgene, with lox-P sites flanking the alternatively spliced exons 5a and 5b of the t-SNARE (target membrane soluble N-ethylmaleimide-sensitive factor attachment protein (SNAP) receptor) gene *Snap25*. Cre-dependent excision of exon 5a/5b leads to a reduced length gene transcript and non-detectable levels of SNAP25 protein, and cessation of Ca^2+^-dependent evoked synaptic vesicle release^15^. Because this chronic t-SNARE disruption allows cortex-wide silencing of selected cell types while avoiding a mechanical manipulation of cortex, which is known to affect the expression of SWA^34^, we opted for this silencing method. In addition, our choice of this mouse model was guided by previous neurodevelopmental studies on the effects of disrupted evoked neurotransmitter release through ablation of SNARE proteins^19,35,36^ as well as by neuroanatomical work conducted in the Rbp4-Cre;Ai14;Snap25^fl/fl^ mouse line^15^. These studies consistently report that brain development, in particular cortical layering and axonal pathfinding, is unaffected by ablation of SNAP25^15,19^. Moreover, as sleep homeostasis occurs on a timescale of minutes to hours, is most likely a distributed process^33,37^, and it is not yet known whether specific areas of the cortex are more involved than others, we considered the Rbp4-Cre;Ai14;Snap25^fl/fl^ mouse model most suitable for the aims of this study^16^. Spontaneous behaviour appeared indistinguishable between genotypes, but conditional knockout mice have a lower body weight compared to Cre-negative controls (cortical SNAP25-ablated: 21.3±0.6 g, controls: 24.4±0.6 g, t(18)=2.94, p=0.009), as reported previously^15^.

### Chronic electrophysiological recordings

EEG/EMG implants were performed in 7 wild type C57BL/6 mice (WT, age at baseline recording 125±8 days), 12 Rbp4-Cre;Ai14;Snap25^fl/fl^ mice (cKO, age at baseline recording 90±5 days) and 8 Cre-negative littermates (CTR, age at baseline recording 85±4 days) under isoflurane anaesthesia as described previously^38^. For analysis of sleep architecture based on EEG/EMG recordings, 9 cKO and 6 CTR mice were included. EEG analysis of frontal and occipital spectra was conducted in 8 cKO and 5 CTR mice. Laminar LFP and MUA could be obtained across cortical layers in primary motor cortex (+ 1.1 mm AP (anterior), - 1.75 mm ML (left), tilt -15° (left)) of 7 WT, 5 cKO, and 5 CTR mice. All laminar recordings were performed using 16-channel silicon probes (NeuroNexus Technologies Inc., Ann Arbor, MI, USA; model: A1x16-3mm-100-703-Z16) with a spacing of 100 μm between individual channels. All experiments were conducted in young adult male mice, because oestrous cycle, development, and aging affect sleep.

All mice implanted for electrophysiological recordings were housed individually in open cages before surgery and in individually ventilated cages during a recovery period of about one week after surgery. For sleep recordings, mice were transferred to separate custom-made Plexiglas cages (20.3 × 32 × 35 cm), which were placed in sound-attenuated and light-controlled Faraday chambers (Campden Instruments, Loughborough, UK), with each chamber fitting two cages. Animals were allowed free access to food pellets and water at all times and underwent daily health inspection. A 12:12 h light:dark cycle (lights on at 9 am, light levels 120–180 lux) was implemented, temperature maintained at around 22 ± 2°C, and humidity kept around 50 ± 20%.

After an acclimatization period of at least 3 days during which animals were habituated to the tethered recording conditions, a 24 h period of continuous recording starting at light onset was performed on a designated baseline day. On the subsequent day, all animals were sleep deprived for 6 h starting at light onset. Sleep deprivation was performed during the circadian period when mice are typically asleep and thus the homeostatic response to sleep loss can be most reliably elicited^21^. At light onset, recording chambers were opened, the nesting material removed, and novel objects placed into the mouse cages to encourage exploratory behaviour. Experimenters continuously observed the mice and exchanged the provided objects for new objects when mice stopped exploring. At the end of the 6 h sleep deprivation, all objects were removed, the nesting material returned, and the recording chambers closed. Sleep deprivation was successful in both genotypes, as only a minimal amount of time (cKO: 1.84±0.75 %, CTR: 1.39±0.32 %, p=0.86 Mann-Whitney U test) was spent asleep during the 6-h interval when the mice were kept awake by providing novel objects.

**Figure 1:**
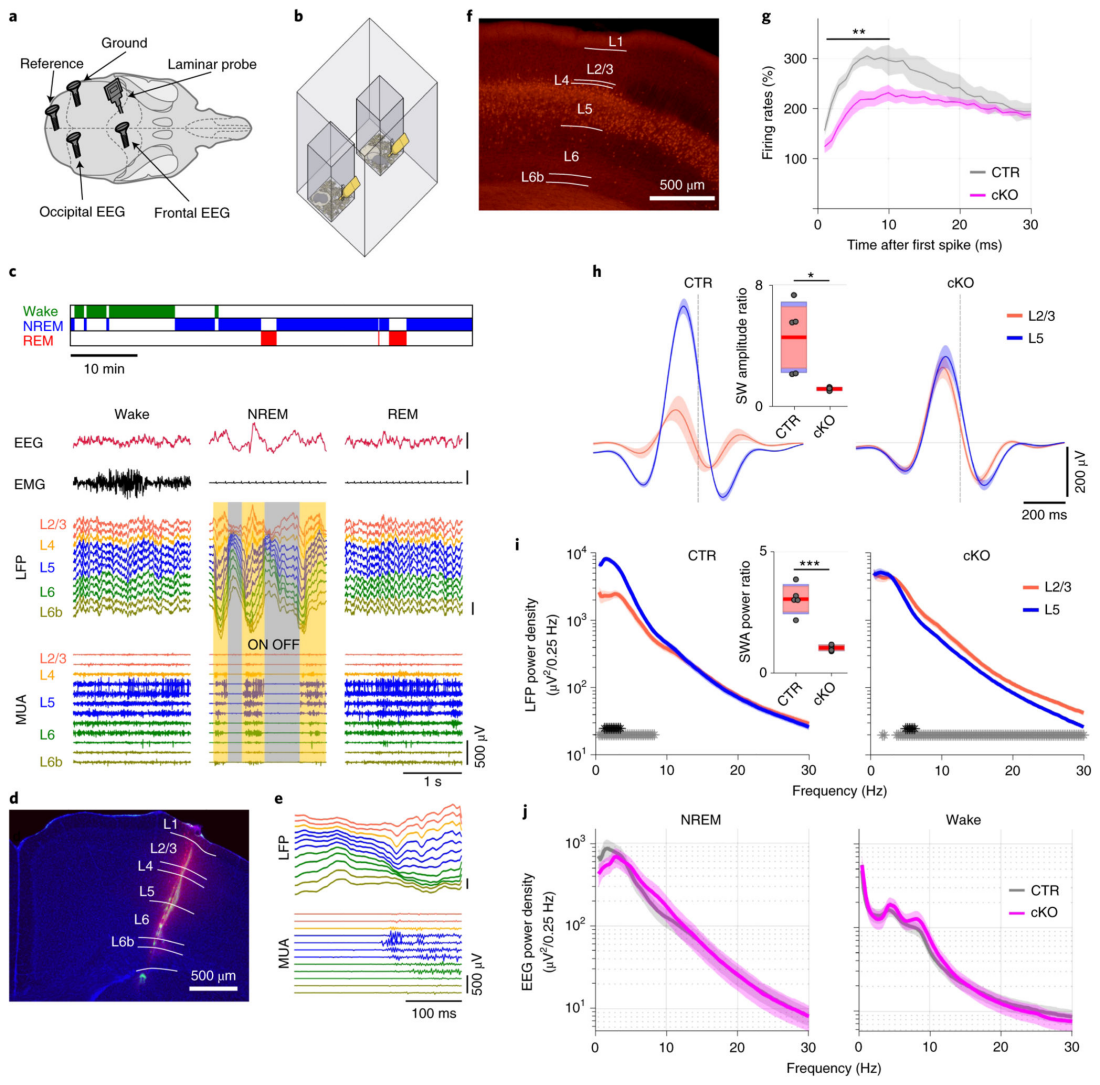
Cortical recordings in freely moving mice implicate layer 5 in the generation of slow waves during NREM sleep. a) EEG/LFP electrode positions. b) Schematic of recording setup. c) Representative hypnogram with corresponding EEG, EMG, LFP, and MUA traces below. Neuronal ON and OFF periods during NREM sleep are highlighted. Scale bars on y-axis: 500μV. d) Insertion tract of a laminar probe. DAPI counterstained (blue) coronal brain section with DiI trace (red). e) Representative OFF-ON transition. Channel assignment and colour coding correspond to panel (c). Scale bars on y-axis: 500μV. f) Neocortical Cre-expression under the Rbp4-Cre promoter. g) Neuronal activity in layer 5 (L5) at OFF-ON transitions in cKO and CTR animals relative to NREM sleep average (*F*(29,232) = 4.326, *p* < 0.001, mixed ANOVA). h) Average LFP slow wave in layers 2/3 (L2/3) and 5 aligned to the OFF-ON transition (vertical dashed line) in cKO and CTR mice (*F*(1,8) = 95.172, *p* < 0.001, mixed ANOVA). Inset: slow wave (SW) amplitude ratio between L2/3 and L5. i) LFP spectra of L2/3 and L5 of cKO and CTR mice during NREM sleep. Asterisks indicate frequency bins with significant differences in post-hoc comparison before (grey) and after (black) Bonferroni adjustment of α *(F*(1,8) = 114.820, *p* < 0.001, mixed ANOVA). Inset: slow wave activity (SWA) ratio between L2/3 and L5. j) Frontal EEG spectra during NREM sleep and wakefulness (*F*(118,1298) = 1.998, *p* < 0.001, mixed ANOVA). n=5 CTR and n=5 cKO for laminar analysis (panels g-i), n=5 CTR and n=8 cKO for EEG spectral analysis (panel j). Black asterisks indicate post-hoc contrasts with significant differences (*p < 0.05, **p < 0.01, ***p < 0.001). Data in panels g-j are presented as mean ± SEM (shaded areas). Insets in panels h, i represent grouped data including group mean (red line), 95% confidence interval (pink box), and one standard deviation (blue box) with individual data points overlaid. See Supplementary Table 1 for detailed results. cKO: conditional knockout animals. CTR: control animals. DiI: 1,1′-Dioctadecyl-3,3,3′,3′-Tetramethylindocarbocyanine Perchlorate. EEG: electroencephalogram. EMG: electromyogram. L2/3, L5: Neocortical layers 2/3, 5. LFP: local field potentials. MUA: multi unit activity. NREM: non-rapid eye movement sleep. REM: rapid eye movement sleep.

The electrophysiological signals revealed typical signatures of wakefulness and sleep states in both genotypes. As expected, the laminar profile of LFPs and MUAs revealed generally activated patterns during waking and rapid eye movement (REM) sleep. Correspondingly, during non-rapid eye movement (NREM) sleep, we observed depth positive LFP slow waves associated with a generalised suppression of spiking activity across cortical layers (Fig. 1c)^20,39^_._

### Electrophysiological acquisition, data processing, and sleep scoring

#### Electrophysiological in vivo recordings

Data was acquired using the 128 Channel Neurophysiology Recording System (Tucker-Davis Technologies Inc., Alachua, FL, USA) and the electrophysiological recording software Synapse (Tucker-Davis Technologies Inc., Alachua, FL, USA), and saved on a local computer. EEG and EMG signals were continuously recorded, filtered between 0.1 - 100 Hz, and stored at a sampling rate of 305 Hz. Extracellular neuronal activity was continuously recorded at a sampling rate of 25 kHz and filtered between 300 Hz - 5 kHz. Whenever the recorded voltage in an individual laminar channel crossed a manually set threshold indicating putative neuronal firing (at least 2 standard deviations above noise level), 46 samples around the event (0.48 ms before, 1.36 ms after) were stored. Concomitantly with the spike acquisition, LFPs were continuously recorded from the same electrodes and processed with the abovementioned settings for EEG signals.

#### Offline signal processing

EEG, EMG, and LFP signals were resampled at a sampling rate of 256 Hz using custom-made code in Matlab (The MathWorks Inc, Natick, Massachusetts, USA, version v2017a) and converted into the European Data Format (EDF) as previously described^38^. Spike wave forms were further processed using a custom-made Matlab script and events with artefactual wave forms were excluded from further analysis of neuronal activity.

#### Scoring of vigilance states

The software Sleep Sign for Animals (version 3.3.6.1602, SleepSign Kissei Comtec Co., Ltd., Nagano, Japan) was used for sleep scoring. EEG, EMG, and LFP recordings were partitioned into epochs of 4 s. Vigilance states were assigned manually to each recording epoch based on visual inspection of the frontal and occipital EEG derivations in conjunction with the EMG. Epochs with recording artefacts due to gross movements, chewing or external electrostatic noise were assigned to the respective vigilance state but not included in the electrophysiological analysis. Overall 18.8±3.5 % of wake, 0.7±0.4 % of NREM, and 0.9±0.4 % of REM epochs contained artefactual EEG signals across all animals included in the EEG spectral analysis, with no significant difference between genotypes. EEG and LFP power spectra were computed using a fast Fourier transform routine (Hanning window) with a 0.25 Hz resolution and exported in the frequency range between 0 and 30 Hz for spectral analysis.

### Non-invasive measurement of home cage activity for circadian phenotyping

Circadian characteristics were assessed in a separate cohort of 10 cKO (age at baseline recording 84±1 days) and 7 CTR mice (age at baseline recording 83±1 days). These mice were housed individually and placed under a passive infrared motion detector that continuously recorded activity in time intervals of 100 ms. Individual readings were pooled into one minute bins for all analyses as it has previously been validated that periods of inactivity of >40 s provide a reliable measure of behaviourally defined sleep^40^. Mice were housed in a stable 12:12 h light:dark cycle for at least 7 days before being released into constant darkness. The circadian period and amplitude were assessed over an interval of 7-10 days in the constant dark condition by ChiSquare periodogram analysis using ActogramJ^41^. After at least 10 days in constant darkness, all mice were exposed to a 2h light pulse provided around Circadian Time 13.5 h. The resulting phase delay of the circadian rhythm was quantified in all animals by extrapolating the onsets of activity to the day of the light pulse^24^.

### Experimental design and statistical analyses

Sample sizes were initially based on previous studies^6,21^. Power calculations were performed after acquisition of pilot data for the Wellcome Trust grant proposal 203971/Z/16/A indicated an effect size of d = 2.75 for the main outcome parameter NREM sleep time over 24 hours. Applying an intended power of 0.9 and an alpha-error probability of 0.01, the power calculations confirmed the initial sample size estimate of n=8 animals per genotype considering an attrition rate of 25%. Animals were retrieved from the breeder colony upon availability. Based on the breeding scheme we expected that male Rbp4-Cre;Ai14;Snap25^fl/fl^ mice only represent on average 1/8 of the animals in each litter. As soon as animals of the desired genotype were available, they were included in the study and the recording capacities were filled with randomly selected Cre-negative controls from the colony, preferably littermates. Data were analysed using MATLAB (version R2017b; The MathWorks Inc, Natick, MA, USA), SAS JMP (version 7.0; SAS Institute Inc. Cary, NC, USA) and IBM SPSS Statistics for Windows (version 25.0; IBM Corp., Armonk, N.Y., USA). Reported averages are mean±s.e.m. Analyses of variance (ANOVAs) were performed as described in Howell and Lacroix (2012)^42^. To examine potential differences in firing dynamics between superficial versus deep cortical layers in WT animals, repeated-measures ANOVAs were conducted with cortical layers (layer 2/3 versus layer 5) and time bins as within-subject factors. To examine potential differences between genotypes, split-plot ANOVAs were conducted; genotype (CTR versus cKO animals) was entered as a between-subject factor, whereas time bins, EEG spectral bins, and vigilance states (wake, NREM, and REM) were entered as within-subject factors. α = 0.05 was adopted for main effects and interactions. Significant two-way interaction terms were followed up with Bonferroni-adjusted post hoc comparisons with αadjusted = 0.05/k, where k represents the total number of pairwise comparisons made^43^. In ANOVAs with multiple time points, the total number of post hoc comparisons was minimised in each case, and hence controlling for the increase in familywise error rate, by pooling across multiple time bins (e.g., 0–10 ms, 11–20 ms, and 21–30 ms in Fig. 1g). For spectral analysis, EEG/LFP power spectra of individual animals were log-transformed before hypothesis testing. Bonferroni correction was not applied for post hoc comparison of spectral data because ANOVAs consisting of 119 EEG spectral bins would be too conservative and reduce statistical power^44^; thus, α was kept at 0.05 in these cases. In the summary of statistical methods and results (Supplementary Table 1) we report frequency bins with significant differences in post-hoc comparison before (α_uncorrected_) and after Bonferroni adjustment of α (α_corrected_). In all figure panels illustrating EEG or LFP spectrograms, frequency bins for which the resulting post-hoc comparison was significant after Bonferroni adjustment are highlighted with black asterisks; bins only significant before Bonferroni adjustment are highlighted with grey asterisks to indicate areas of interest bearing in mind that no correction for multiple comparisons was applied. In the circadian analysis, a multivariate ANOVA (MANOVA) was conducted to examine if the linear combination of multiple circadian response variables (period length, periodogram power, and phase shift) differed between genotypes^45^. Data distribution was assessed graphically and formally tested if normality and equal variances could not be assumed. Greenhouse-Geisser correction was used when the assumption of sphericity was violated (Mauchly’s test of sphericity, p<0.05). Mann-Whitney U tests were performed for main analyses instead of one-way ANOVAs if the assumption of normality was violated (Shapiro-Wilk test of normality, p<0.05). The statistical methods and results from each individual analysis are listed in Supplementary Table 1. In all figures, significance levels are indicated with black asterisks: ‘*’ for 0.05 ≥ p > 0.01; ‘**’ for 0.01 ≥ p > 0.001; ‘***’ for 0.001 ≥ p. Grey asterisks indicate post-hoc comparisons with *P* < 0.05, which do not reach significance after Bonferroni-correction for multiple comparisons. Panels representing grouped data of durations spent in specific vigilance states show group mean (red line), 95% confidence interval (pink box), and one standard deviation (blue box) with individual data points overlaid; these plots were generated using the MATLAB functions notBoxPlot (Rob Campbell (2019). notBoxPlot (https://www.github.com/raacampbell/notBoxPlot), GitHub. Retrieved June 15, 2019) and RGB (Kristjan Jonasson (2019). RGB triple of color name, version 2 (https://www.mathworks.com/matlabcentral/fileexchange/24497-rgb-triple-of-color-name-version-2), MATLAB Central File Exchange. Retrieved June 15, 2019). For key analyses reported in the main text, the effect sizes are reported as Cohen’s d calculated using the MATLAB function computeCohen_d (Ruggero G. Bettinardi (2021). computeCohen_d(x1, x2, varargin) (https://www.mathworks.com/matlabcentral/fileexchange/62957-computecohen_d-x1-x2-varargin), MATLAB Central File Exchange. Retrieved October 4, 2020).

### Electrophysiological criteria for analysis of vigilance state episodes and OFF periods

**Extended Data Fig. 1:**
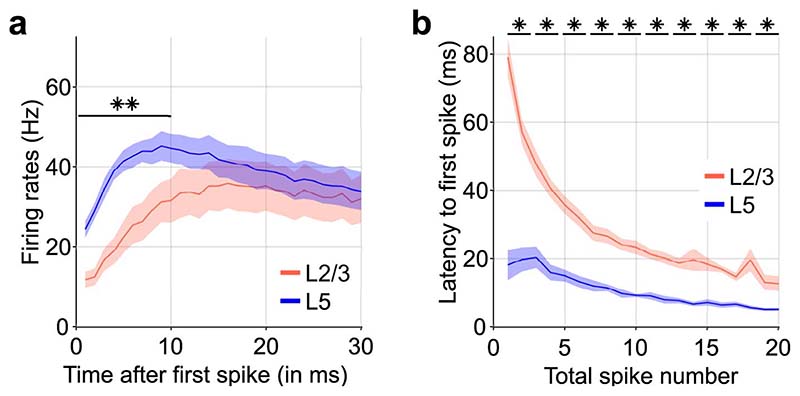
Neuronal dynamics around OFF-ON transitions imply a leading role of layer 5 in population activity. a) Average neuronal firing activity at the transitions from population OFF to ON periods during baseline NREM sleep (*F*(29,174) = 9.412, *p* < 0.001, two-factor repeated measures ANOVA). Note that firing rates are higher in layer 5 during the first 10ms. b) Latency to the first spike for matched spike numbers during the first 200ms of an ON period mice (*F*(1,6) 86.301, *p* < 0.001, two-factor repeated-measures ANOVA). Note that the latency to the first spike is shorter in layer 5 irrespective of the total number of spikes in a given ON period. n=7 wild type (C57L/6). Black asterisks indicate post-hoc contrasts with significant differences (*p < 0.05, **p < 0.01, ***p < 0.001). Data in panels a, b are presented as mean ± SEM (shaded areas). See Supplementary Table 1 for detailed results. L2/3, L5: Neocortical layers 2/3, 5. NREM: non-rapid eye movement sleep.

**Figure 2:**
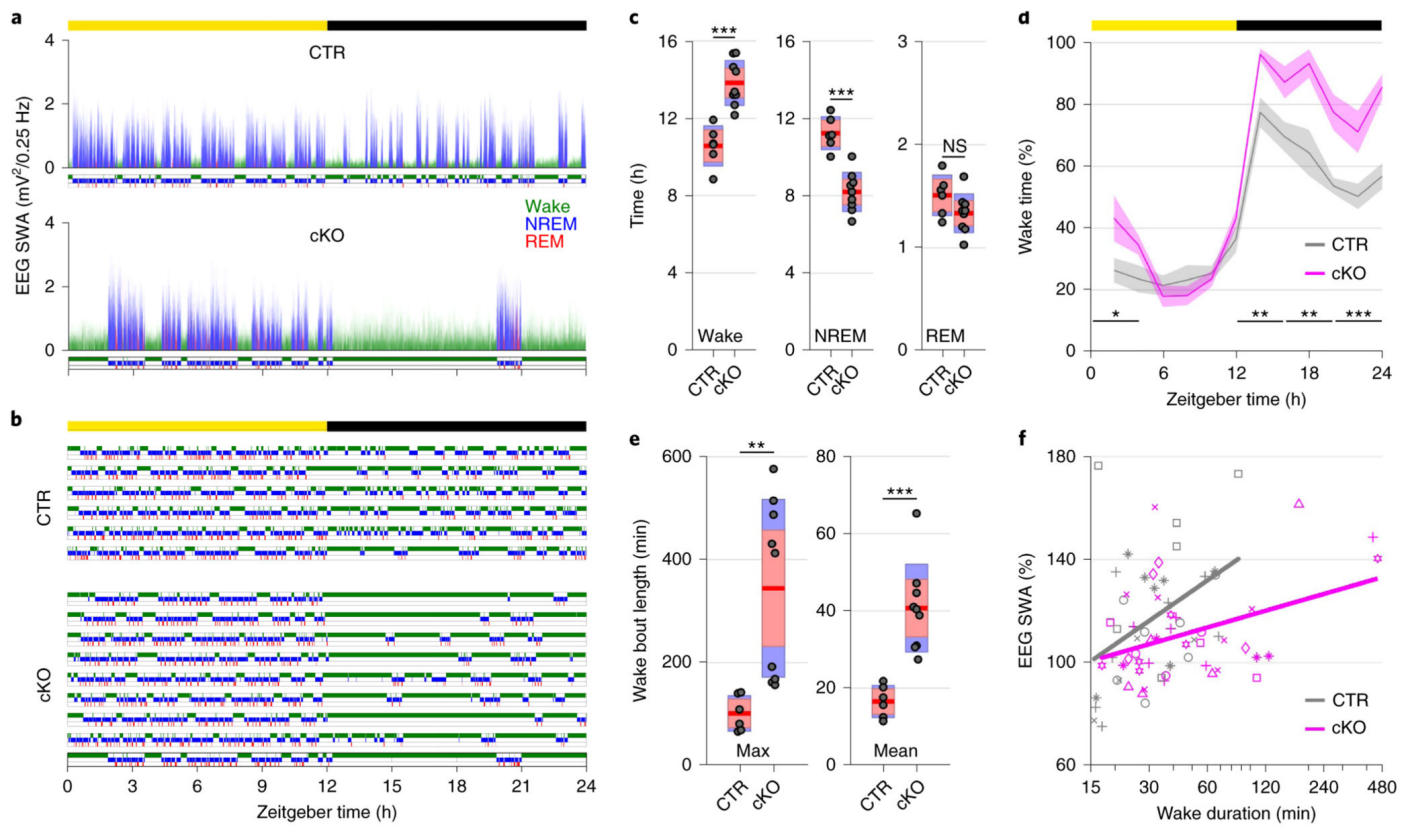
Selective cortical SNAP25 ablation alters sleep architecture. a) Hypnogram and EEG slow wave activity (0.5-4.0Hz, 4s epochs) of one representative animal from each genotype in undisturbed 24h baseline recordings. b) Individual hypnograms for CTR and cKO animals under undisturbed baseline conditions. Note the increased amount of wakefulness and long wake episodes in cKOs. a&b colour coding: wake=green, NREM=blue, REM=red. c) Time spent in vigilance states (wake, NREM, and REM) during 24h baseline recordings (*F*(1.056,13.732) = 33.008, *p* < 0.001, mixed ANOVA). d) Time course of wakefulness over 24h baseline recordings. (*F*(1,13) = 30.804, *p* < 0.001, mixed ANOVA). e) Maximum and mean duration of all spontaneous wake episodes over the 2-day recording period, excluding the 6 h sleep deprivation (*F*(1,13) = 11.326, *p* = 0.005 for maximum duration; *F*(1,13) = 24.392, *p* < 0.001 for mean duration, two one-way ANOVAs). f) Relationship between wake duration and relative NREM SWA in the frontal EEG derivation during the baseline day. Individual animals are represented with different symbols. n=6 CTR and n=9 cKO for vigilance state analysis (panels c, d, e), n=5 CTR and n=8 cKO for EEG spectral analysis (panel f). Black asterisks indicate post-hoc contrasts with significant differences (*P < 0.05, **P < 0.01, ***P < 0.001), grey asterisk indicates post-hoc comparison with *P* < 0.05, which does not reach significance after Bonferroni correction for multiple comparisons. Data in panel d are presented as mean ±SEM (shaded areas). Yellow and black bars above panels a, b and d indicate light and dark periods, respectively. Panels c and e represent grouped data including group mean (red line), 95% confidence interval (pink box), and one standard deviation (blue box) with individual data points overlaid. See Supplementary Table 1 for detailed results. BL: baseline. cKO: conditional knockout animals. CTR: control animals. EEG: electroencephalogram. NREM: non-rapid eye movement sleep. REM: rapid eye movement sleep. SD: sleep deprivation. ZT: zeitgeber time.

For analyses of mean and maximum duration of sustained wake episodes in the EEG dataset (Fig. 2e), we included wake episodes, which were at least 1 min long allowing brief intrusions of sleep of 1 min or less. For the analysis of mean duration of NREM episodes, we included NREM episodes, which were at least 1 min long allowing brief intrusions of REM sleep or brief awakenings of 1 min or less (Extended Data Fig. 7). To investigate the change in NREM SWA across prolonged wake episodes under undisturbed conditions, we used the EEG dataset from the baseline day and included consolidated periods of waking lasting at least 15 min, whereby short episodes of sleep <1 min were not considered as interruptions. We then performed analyses of NREM sleep SWA in the 15-min time window immediately preceding and following prolonged (>15 min) wake episodes, if both time windows included at least 10 min of artefact-free NREM sleep and no more than 3 min of wakefulness (Fig. 2f). Population OFF periods were defined as periods of total neuronal silence across all electrodes, which lasted at least 50 ms and no more than 4000 ms. Subsequently, the top 20% longest OFF periods were included for final analyses (Fig. 1g, h and Extended Data Fig. 1). The latency to the first spike after the population OFF-ON transition was calculated separately for MUA recorded in layers 2/3 and layer 5. Only ON periods with at least 1 spike in each of the layers occurring within the first 200 ms were included in this analysis (Extended Data Fig. 1).

### Histological assessment of laminar probe depth

The tips of laminar implants were stained before surgery with the orange-red fluorescent membrane stain Dil ^®^ (1,1′-Dioctadecyl-3,3,3′,3′-Tetramethylindocarbocyanine Perchlorate; Thermo Fisher Scientific, Waltham, MA, USA) by immersion of the electrode shank into a 20 mg/ml solution (50/50% acetone/methanol) for later histological assessment of the electrode position^46^. After completion of the experiments, microlesions of selected channels on the laminar probe were performed under terminal pentobarbital anaesthesia using the electroplating device NanoZ™ (White Matter LLC, Seattle, WA, USA) applying 10 mA direct current for 25 s to each respective channel. Immediately following microlesioning, mice were perfused with 0.1M phosphate buffered saline (0.9 %) (PBS) followed by 4 % paraformaldehyde in PBS for tissue preservation. A vibrating microtome (Leica VT1000S) was used to section the brains into 50 μm coronal slices. Fluorescent staining was performed with 4′,6-diamidino-2-phenylindole (DAPI). After fluorescence microscopy, implantation sites were mapped using a mouse brain atlas^47^ and the depth of the laminar implant was assessed measuring the distance between cortical surface and the electrical current induced tissue microlesions. The histological assessment of the insertion tract was performed in all animals, which were implanted with laminar probes. Structural integrity of cortex and the position of the laminar implant similar to those shown in the representative examples (Fig. 1d and Supplementary Figure 2b) were found in each individual animal included in the electrophysiological analysis of laminar data. ImageJ (version 1.52a) was used to merge fluorescence images and add scale bars^48^. All figures were created using Inkscape (version 1.0.2, Inkscape Project. (2020). Inkscape. Retrieved from https://inkscape.org ).

### Histological assessment of Cre-expression in cortex, dentate gyrus, and hypothalamus

Coronal brain sections from six Rbp4-Cre;Ai14;Snap25^fl/+^ mice were prepared using the same procedure as outlined above for the histological assessment of the laminar probe depth. Laser scanning confocal microscope images were acquired (Zeiss LSM710) and image stacks created to assess the cellular morphology across the z-plane of the brain section. The cytoplasmic tdTomato expressed from the *Ai14* reporter gene allows reliable identification of Cre+ neurons based on native tdTomato fluorescence^15,49^. The native fluorescence is detectable even in thin neurites and synapses, which can be used to visualise the dendritic and axonal morphology of labelled neurons. The histological assessment of Cre-expression was performed in six Rbp4-Cre;Ai14;Snap25fl/+ mice and the results across all animals were similar to those presented in the representative images.

### Immunohistochemistry to determine lateral hypothalamic cell identity

To determine whether Cre+ neurons in the lateral hypothalamus express melanin concentrating hormone (MCH) or orexin/hypocretin (Hcrt), coronal sections from three Rbp4-Cre;Ai14 were prepared as described above. Before counterstaining with DAPI, the sections were stained with rabbit anti-MCH antibody (1:2000; H-070-47; Phoenix Pharmaceuticals) or rabbit anti-Hcrt antibody (1:500, kind gift from Anthony Van den Pol, Yale University). Briefly, sections were incubated in blocking solution (3% or 10% donkey serum [Hcrt or MCH respectively] and 0.3% Triton-X100 in 0.1M PBS) for one hour before incubation with the primary antibody in blocking solution at 4°C overnight (MCH) or for 72h (Hcrt). The primary antibody was revealed by incubating with donkey anti-rabbit-AlexaFluor488 (1:500) antibody in blocking solution for two hours at room temperature. Subsequently, epifluorescence and confocal imaging was performed as described above.

## Extended Data

**Extended Data Fig. 2:**
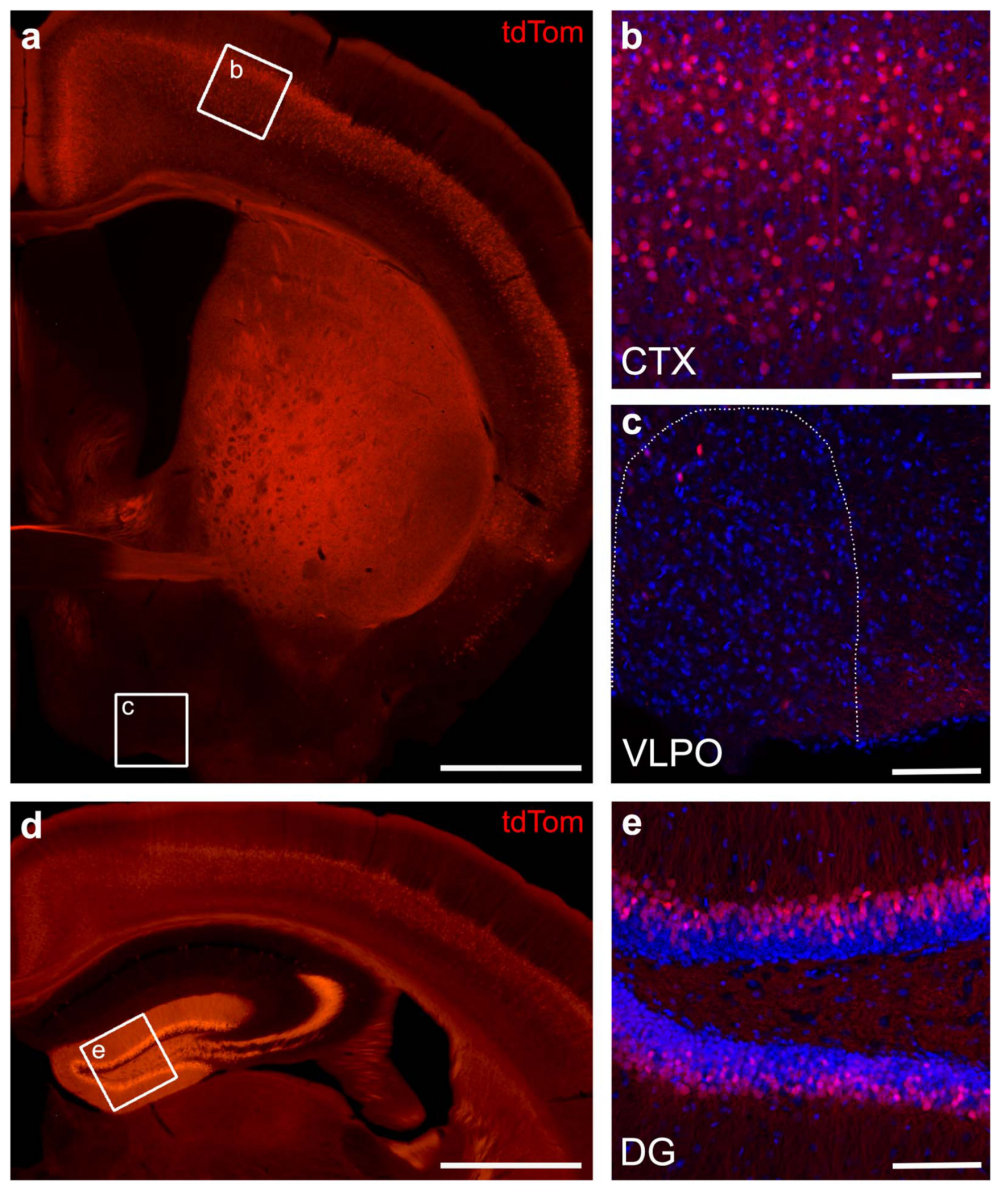
Comparison of Cre-expression in the Rbp4-Cre mouse line between neocortical layer 5, dentate gyrus and hypothalamus. a) Coronal section of an Rbp4-Cre;Ai14;Snap25^fl/+^ mouse brain indicating areas, which were further examined for Cre-expression using confocal imaging of DAPI stained slices. b,c) Laser scanning confocal microscope images from neocortex (CTX, b) and ventrolateral preoptic hypothalamus (VLPO, c) of DAPI stained (blue) sections, showing the distribution of tdTomato+ cells in the two regions. The VPLO region is outlined with a white, dotted line. Cell counts on corresponding coronal sections in three brains revealed that 20.53±0.98% (480/2342) of cortical L5 cells were tdTomato+, while only 1.15±0.40% (35/3006) of hypothalamic cells expressed the red fluorescent indicator. d) Coronal section of an Rbp4-Cre;Ai14;Snap25^fl/+^ mouse brain indicating the area of the dentate gyrus which was further examined for Cre-expression. e) Laser scanning confocal microscope image of dentate gyrus (DG) in a DAPI stained (blue) section. TdTomato+ cells were quantified in both the top and bottom blades of DG in three images, each from three different brains, and comprise 39.39±3.72% of cells in the granule layer. As evident from the boxed regions in (a,d), the tdTomato+ cells in different brain regions vary in their fluorescence intensity, therefore the images in panels (b,c,e) were acquired with settings optimised to show the tdTomato+ cells in each brain region. CTX: neocortex. DAPI: 4′,6-diamidino-2-phenylindole. DG: dentate gyrus. VLPO: ventrolateral preoptic hypothalamus. Scale bars: 1mm (a,d), 100 μm (b,c,e)

**Extended Data Fig. 3:**
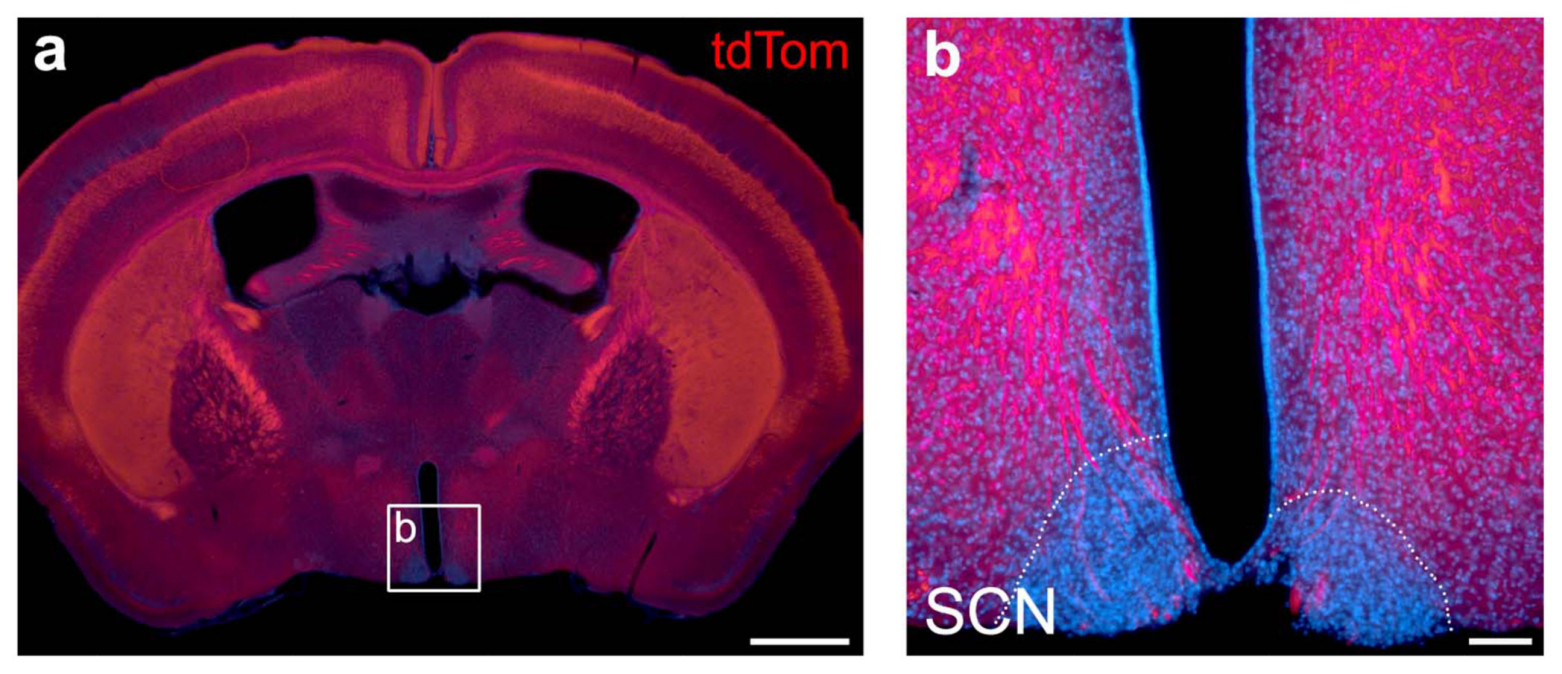
The suprachiasmatic nucleus of the hypothalamus is void of Cre+ cells and spared of fibre tracts in the Rbp4-Cre driver line. (a) Epifluorescence image of an Rbp4-Cre;Ai14;Snap25fl/+ brain section at the level of the suprachiasmatic nucleus (SCN). The section was counterstained with DAPI (blue). Box indicates approximate region from which image in (b) was taken. (b) High-magnification epifluorescence image of the SCN region. Rbp4-Cre;Ai14 axons are shown in red, cell nuclei stained with DAPI in blue. Note that there are no Cre+ cells located within the SCN (outlined with white dotted lines), and very few of the dense axon bundles pass through the SCN. DAPI: 4′,6-diamidino-2-phenylindole. SCN: suprachiasmatic nucleus. Scale bars: 1mm (a), 100 μ⍰m (b)

**Extended Data Fig. 4:**
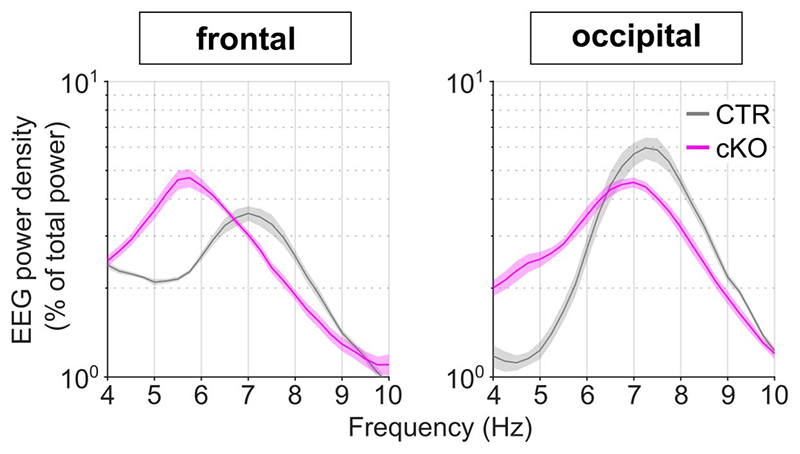
No overlap between orexin or melanin concentrating hormone-expressing cells with Rbp4-Cre+ cells but dense fibre tracts in lateral hypothalamus. (a) Epifluorescence image of an Rbp4-Cre;Ai14;Snap25^fl/+^ brain hemisection at the level of the lateral hypothalamic area (LH), stained for melanin concentrating hormone (MCH) in green. Boxes indicate approximate regions from which images in (b,c) were taken. (b,c) Laser scanning confocal microscope images of two representative sections of LH of the same brain as shown in (a) stained for MCH (b) or orexin/hypocretin (Hcrt; c). Rbp4-Cre;Ai14 cells and processes are shown in red, and nuclei are counterstained with DAPI (blue). Note that no MCH+ cell was tdTom+ (n=3 brains, 692 MCH+ cells), and no Hcrt+ cell was tdTom+ (n=3 brains, 469 Hcrt+ cells). Note the dense fine fibres surrounding cell bodies in LH, consistent with an axonal terminal field in that region. DAPI: 4′,6-diamidino-2-phenylindole. Hcrt: orexin/hypocretin. MCH: melanin concentrating hormone. tdTom: tdTomato. Scale bars: 1mm (a), 100 μm (b,c)

**Extended Data Fig. 5:**
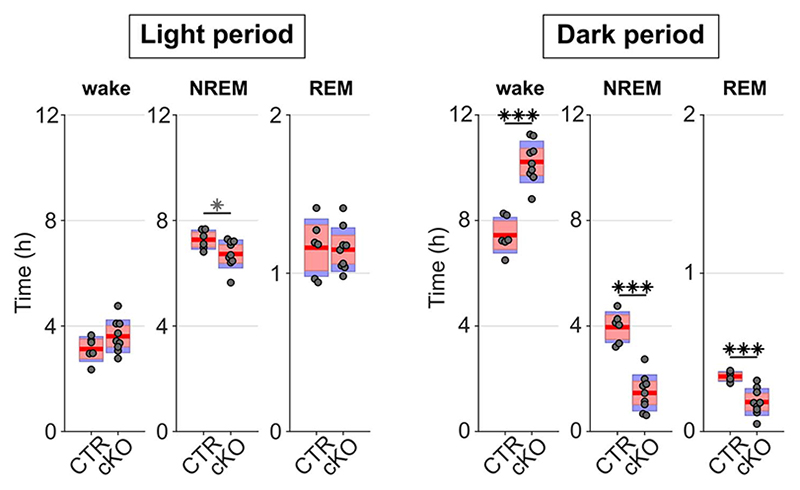
The theta peak during REM sleep is shifted towards lower frequencies in cortical SNAP25-ablated mice. EEG spectral power in the frequency range 4-10 Hz normalised to the mean spectral power over the entire EEG spectrum (0.5 – 30 Hz) during REM sleep on the baseline day. Note that peak theta activity is shifted towards lower frequencies in cKOs compared to CTRs in both the frontal and occipital EEG derivations. n=5 CTR and n=8 cKO for EEG spectral analysis. Data in are presented as mean ± SEM (shaded areas). See Supplementary Table 1 for detailed results. cKO: conditional knockout animals. CTR: control animals. EEG: Electroencephalogram. REM: Rapid eye movement sleep.

**Extended Data Fig. 6:**
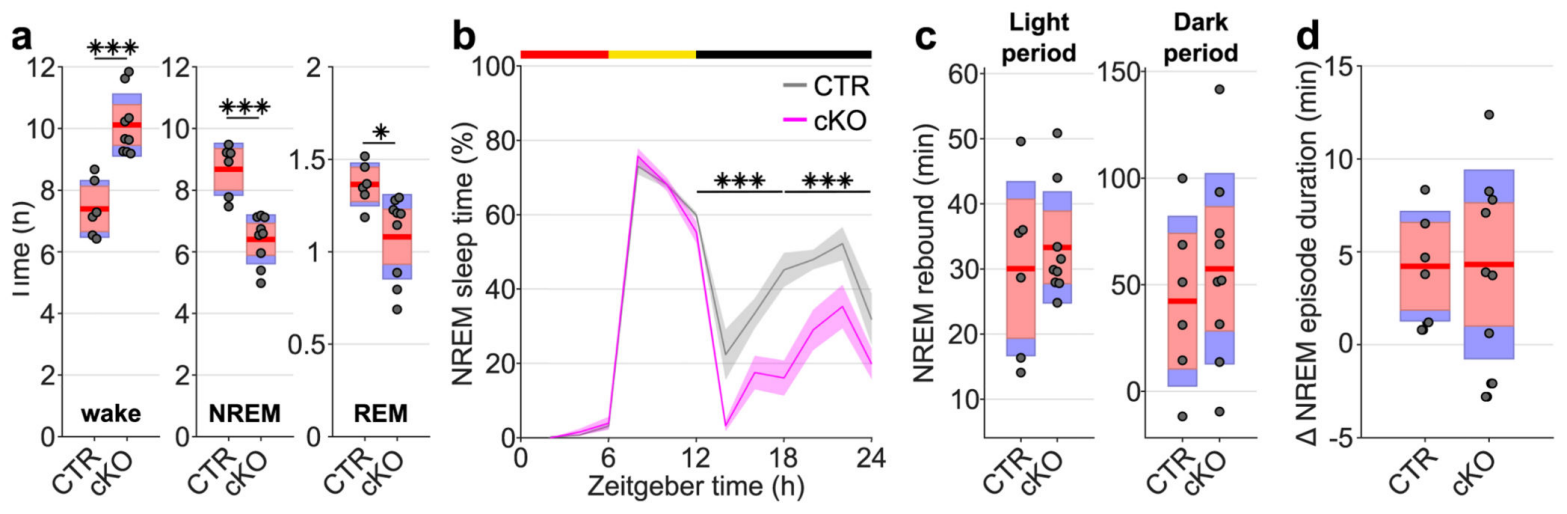
Genotype differences in the amount of time spent in wake, NREM, and REM sleep during undisturbed baseline recordings are more pronounced in the dark period. During the light period, the distribution between vigilance states is similar between genotypes with only a trend towards increased wakefulness and reduced NREM and REM sleep, while strong differences occur during the dark period (*F*(1,14) = 36.083, *p* < 0.001, three-way ANOVA). n=6 CTR and n=9 cKO for vigilance state analysis. Black asterisks indicate post-hoc contrasts with significant differences (*p < 0.05, **p < 0.01, ***p < 0.001), grey asterisks indicate post-hoc comparisons with *P* < 0.05, which do not reach significance after Bonferroni correction for multiple comparisons. Data is presented as group mean (red line), 95% confidence interval (pink box), and one standard deviation (blue box) with individual data points overlaid. See Supplementary Table 1 for detailed results. cKO: conditional knockout animals. CTR: control animals. NREM: Non-rapid eye movement sleep. REM: Rapid eye movement sleep.

**Extended Data Fig. 7:**
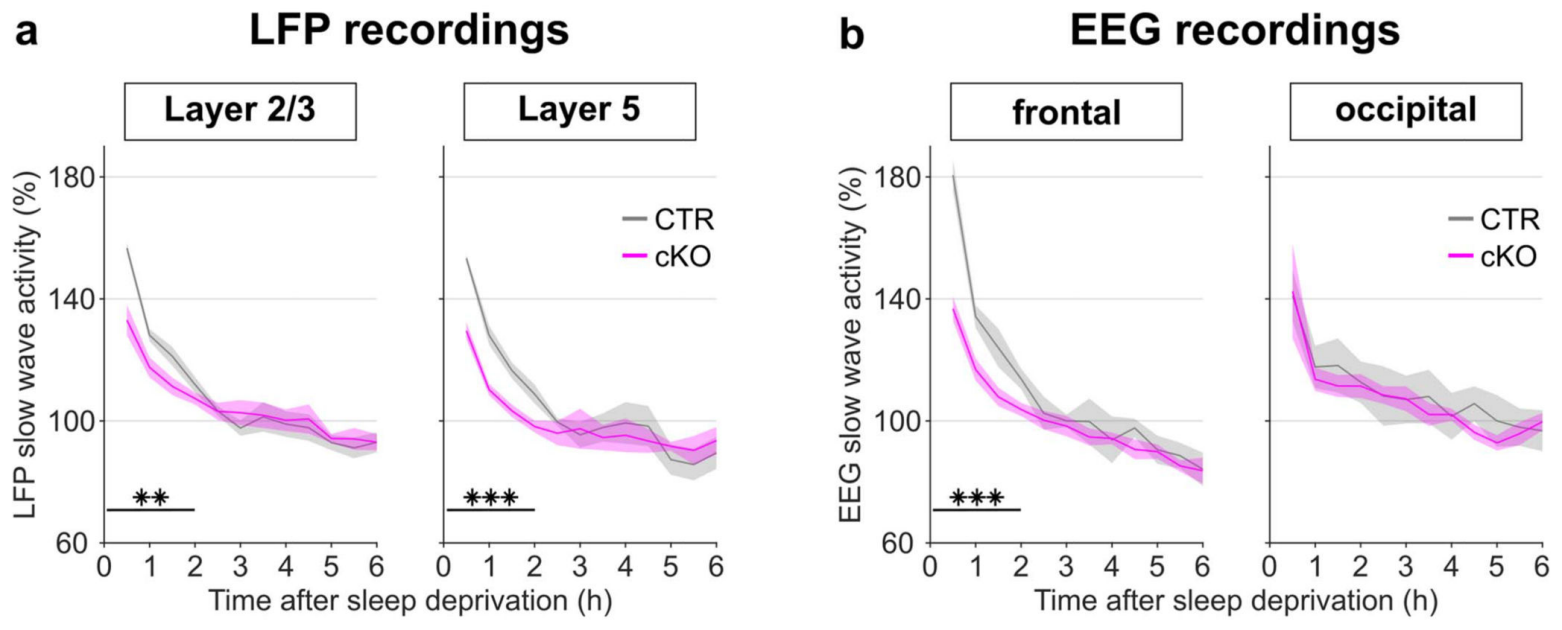
Absolute time in NREM sleep following sleep deprivation is reduced in cortical SNAP25-ablated animals but relative NREM rebound does not differ between genotypes a) Time spent in vigilance states (wake, NREM, and REM) during the 18 h recovery time following sleep deprivation (*F*(1,14) = 27.754, *p* < 0.001, mixed ANOVA). Note that cortical SNAP25-ablated animals (cKO) overall spent more time awake and less time in NREM and REM sleep compared to controls (CTR). b) Time course of NREM sleep on a sleep deprivation day compared between genotypes (*F*(4,54) = 4.222, *p* = 0.004, mixed ANOVA). cKOs sleep less during the entire 12 h dark period following sleep deprivation. c) Rebound of NREM sleep time following sleep deprivation relative to individual baseline values. No differences were observed between genotypes. d) Change in duration of NREM episodes during the first hour after sleep deprivation (ZT6-7 of SD day) relative to the same time window on BL day. n=6 CTR and n=9 cKO for vigilance state analysis. Black asterisks indicate post-hoc contrasts with significant differences (*p < 0.05, **p < 0.01, ***p < 0.001). Data in panels a, c, d is presented as group mean (red line), 95% confidence interval (pink box), and one standard deviation (blue box) with individual data points overlaid. Data in panel b are presented as mean values ± SEM(shaded areas). See Supplementary Table 1 for detailed results. BL: baseline. cKO: conditional knockout animals. CTR: control animals. NREM: Non-rapid eye movement sleep. REM: Rapid eye movement sleep. SD: sleep deprivation. ZT: zeitgeber time.

**Extended Data Fig. 8:**
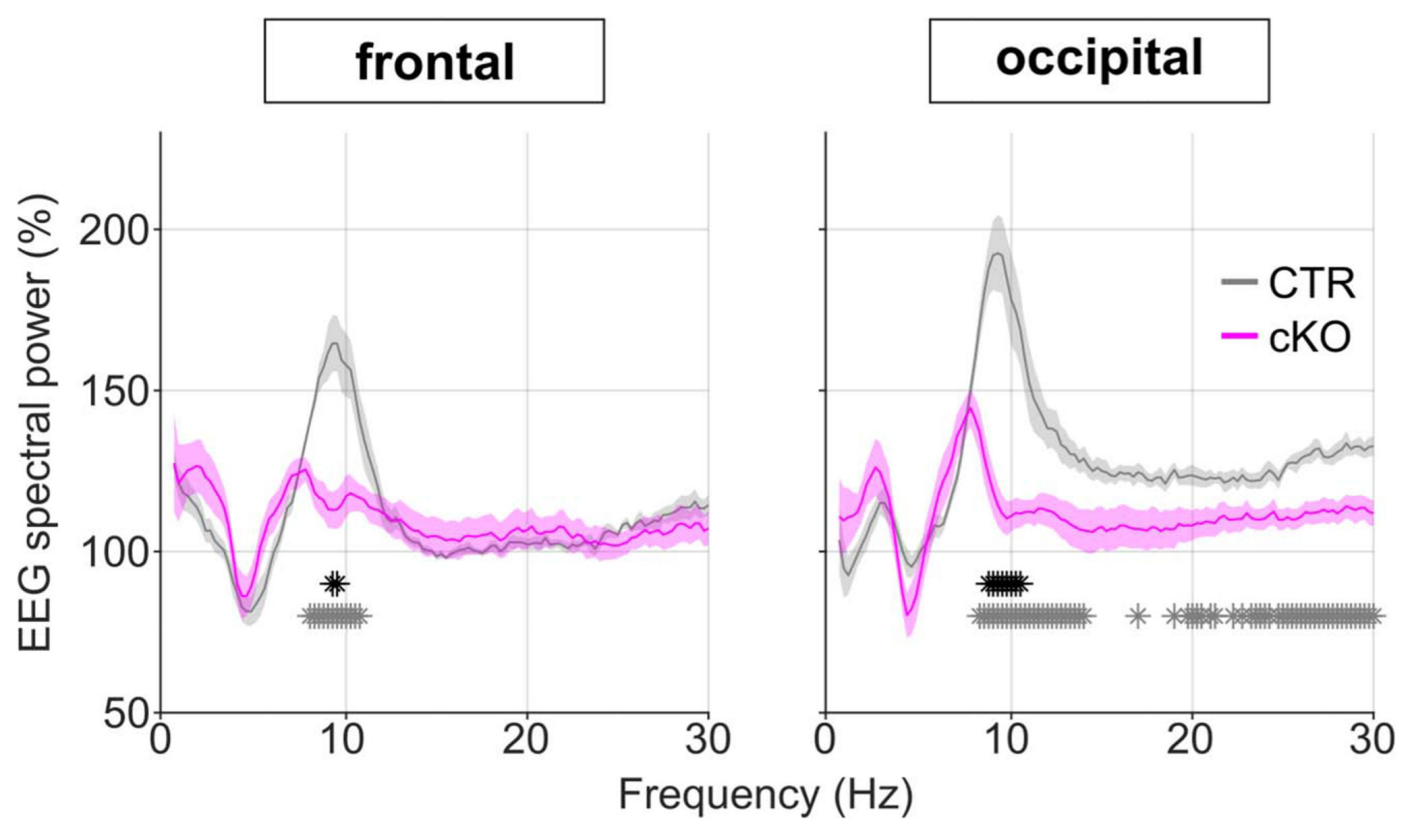
The rebound of slow wave activity after sleep deprivation is specific to cortical areas but not layers. Time course of NREM slow wave activity (SWA) after sleep deprivation (a) in the LFPs from layers 2/3 and 5 in primary motor cortex and (b) in the frontal and occipital EEG derivation. Note that cortical SNAP25-ablated animals (cKO) had lower initial SWA levels in the frontal EEG and LFP recordings across all layers, compared to controls (CTR). n=5 CTR and n=5 cKO for laminar analysis, n=5 CTR and n=8 cKO for EEG spectral analysis. Black asterisks indicate post-hoc contrasts with significant differences (*p < 0.05, **p < 0.01, ***p < 0.001). Data in panels a and b are presented as mean values ± SEM (shaded areas). See Supplementary Table 1 for detailed results. cKO: conditional knockout animals. CTR: control animals.

**Extended Data Fig. 9:**
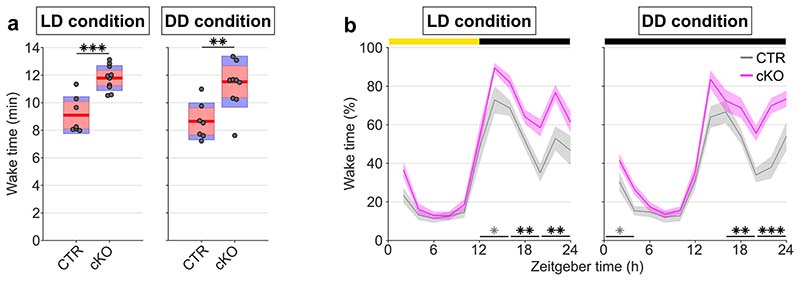
Relative EEG power spectra during sleep deprivation show an attenuated increase in theta activity cortical SNAP25-ablated animals. Wake EEG spectral power during the 6-hour sleep deprivation shown as a frequency bin-wise percentage of 24h baseline values. Note that the expected increase in theta-power during sleep deprivation, which is visible in CTR mice, is severely diminished in cKOs. n=5 CTR and n=8 cKO for EEG spectral analysis. Individual asterisks indicate spectral bins with significant differences in post-hoc comparison before (grey) and after (black) Bonferroni adjustment of α. Data in are presented as mean values ± SEM (shaded areas). See Supplementary Table 1 for detailed results. cKO: conditional knockout animals. CTR: control animals. EEG: electroencephalogram.

**Extended Data Fig. 10:**
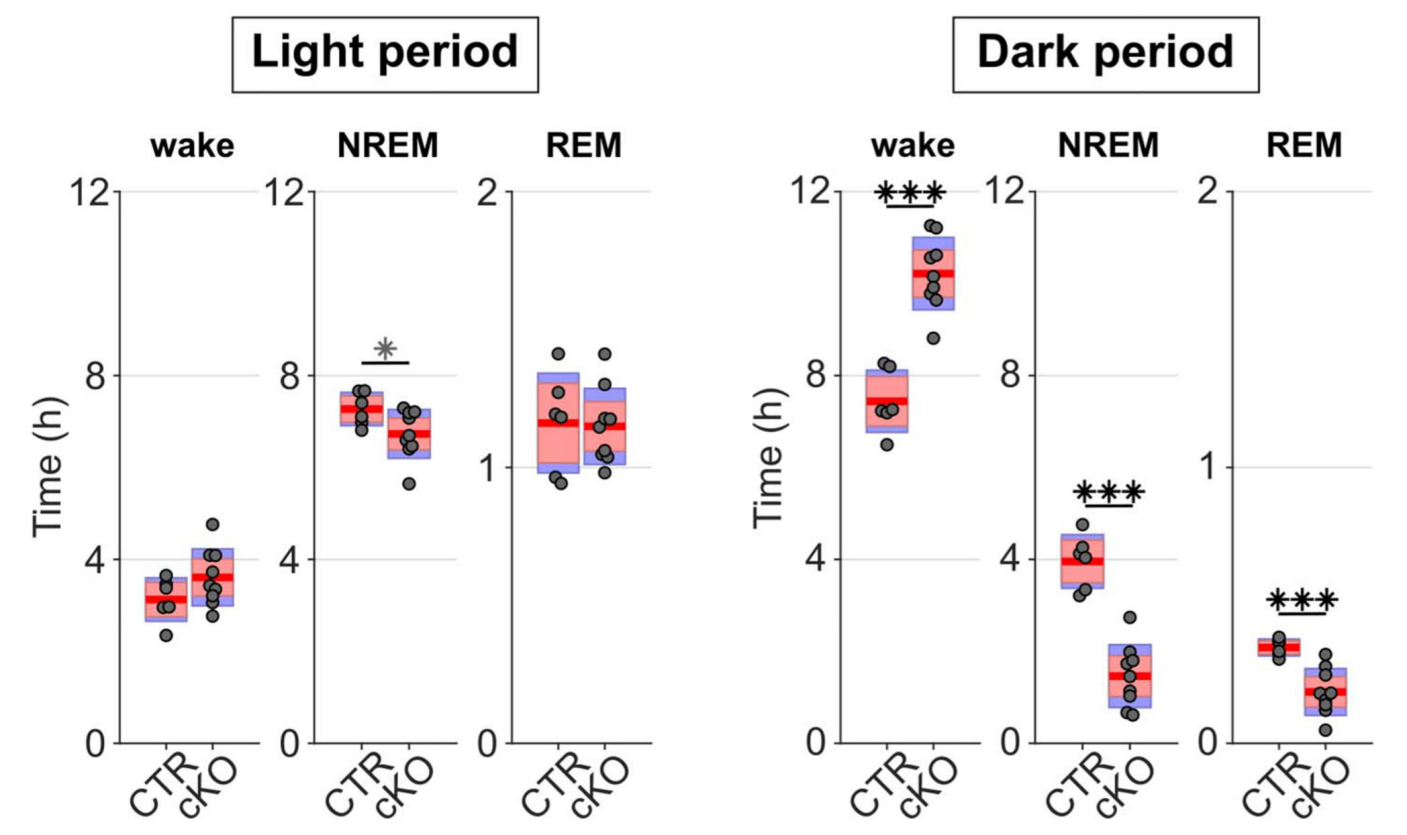
Passive infrared recordings (PIR) show robustness of sleep phenotype to altered light-conditions. a) Wake time estimates averaged over the last 3 days of PIR recordings under 12:12 light-dark (LD) conditions and over the first 3 days of constant darkness (DD) (*F*(1,15) = 18.604, *p* = 0.001, mixed ANOVA). Note that genotype differences in the daily amount of wakefulness persist in the absence of light. b) Time course of wakefulness in LD and DD conditions (*F*(3.801,57.016) = 3.319, *p* = 0.018, mixed ANOVA). n=7 CTR and n=10 for PIR recordings. cKO: conditional knockout animals. Black asterisks indicate post-hoc contrasts with significant differences (*p < 0.05, **p < 0.01, ***p < 0.001), grey asterisks indicate post-hoc comparisons with *P* < 0.05, which do not reach significance after Bonferroni correction for multiple comparisons. Data in panel a is presented as group mean (red line), 95% confidence interval (pink box), and one standard deviation (blue box) with individual data points overlaid. Data in panel b are presented as mean values ± SEM (shaded areas). See Supplementary Table 1 for detailed results. CTR: control animals. LD: light:dark. DD: constant darkness. PIR: passive infrared recordings.

## Supplementary Material

EMS140496-Supinfo

EMS140496_SD-1

EMS140496_SD-2

EMS140496_SD-3

EMS140496_SD_ED-6

EMS140496_SD_ED-1

EMS140496_SD_ED-9

EMS140496_SD_ED-7

EMS140496_SD_ED-5

EMS140496_SD_ED-8

EMS140496_SD_ED-10

EMS140496_Sup_data-3

EMS140496_Sup_data-2

EMS140496_Sup_data-1

reportingsummary

## Figures and Tables

**Figure 3:**
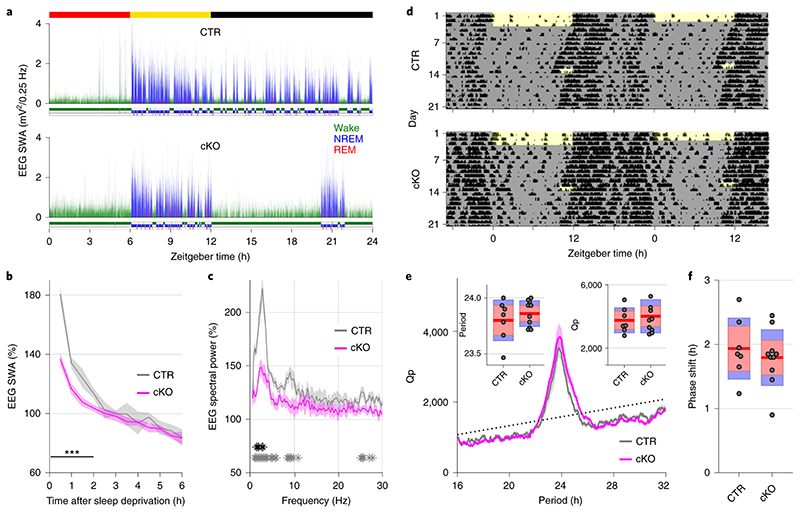
Selective cortical SNAP25 ablation alters homeostatic but not circadian sleep regulation. a-c) Homeostatic response to 6h sleep deprivation at light onset based on EEG spectral analysis. a) Hypnogram and EEG slow wave activity (0.5-4.0Hz, 4s epochs) of one representative mouse from each genotype (red, yellow and black bars indicate sleep deprivation, light and dark periods, respectively). b) Time course of frontal EEG slow wave activity during NREM sleep after sleep deprivation relative to baseline average (*F*(11,121) = 7.561, *p* < 0.001, mixed ANOVA). Note the reduced SWA in cKO compared to CTR mice during the first 2 hours after sleep deprivation. c) NREM sleep spectral power in the frontal derivation during the first 30 minutes after sleep deprivation relative to baseline NREM average (*F*(118,1298) = 4.068, *p* < 0.001, mixed ANOVA). Asterisks indicate frequency bins with significant differences in post-hoc comparison before (grey) and after (black) Bonferroni adjustment of α. d-f) Circadian phenotyping based on passive infrared recordings (PIR). d) Representative actograms of one mouse from each genotype. Day 1-3: 12:12h LD cycle. From day 4: constant darkness protocol. Day 13: phase-delaying 2h light pulse. e) Average Chi-square periodogram. Insets: period (h) and periodogram power (Qp). f) Phase shift in activity onset on the day after the light pulse. No significant differences were observed in any of the assessed circadian characteristics. n=5 CTR and n=8 cKO for EEG spectral analysis (panel b,c), n=7 CTR and n=10 cKO for PIR analysis (panels e,f). Black asterisks indicate post-hoc contrasts with significant differences (*p < 0.05, **p < 0.01, ***p < 0.001). Data in panels b, c, e are presented as mean ± SEM (shaded areas). Panels e and f represent grouped data including group mean (red line), 95% confidence interval (pink box), and one standard deviation (blue box) with individual data points overlaid. See Supplementary Table 1 for detailed results. cKO: conditional knockout animals. CTR: control animals. EEG: electroencephalogram. LD: light:dark. NREM: Non-rapid eye movement sleep. PIR: passive infrared recordings. SWA: Slow wave activity (0.5-4.0Hz).

## Data Availability

Custom-made Matlab code for key analyses is deposited on Figshare (DOI: 10.6084/m9.figshare.14737578). Code used for additional analyses is available from the corresponding authors upon reasonable request.

## Reporting summary

Further information on experimental design and reagents is available in the nature Research Life Sciences Reporting Summary linked to this paper.

## Data availability

Statistical source data for all figures is available for download via the Nature Neuroscience weblink to this paper. Imaging source data is deposited on Figshare (DOI: 10.6084/m9.figshare.14737584). A sample dataset with spectral data and sleep scoring results used to generate key analyses presented in this manuscript is available for download on Figshare (DOI: 10.6084/m9.figshare.14737569). Raw data from electrophysiological and passive infrared recordings are available from the corresponding authors upon reasonable request.

## References

[1] Saper CB, Fuller PM (2017). Wake–sleep circuitry: an overview. Curr Opin Neurobiol.

[2] Liu D, Dan Y (2019). A Motor Theory of Sleep-Wake Control: Arousal-Action Circuit. Annu Rev Neurosci.

[3] Vyazovskiy VV (2009). Cortical Firing and Sleep Homeostasis. Neuron.

[4] Borbély AA, Daan S, Wirz-Justice A, Deboer T (2016). The two-process model of sleep regulation: A reappraisal. J Sleep Res.

[5] Huber R, Felice Ghilardi M, Massimini M, Tononi G (2004). Local sleep and learning. Nature.

[6] Vyazovskiy VV (2011). Local sleep in awake rats. Nature.

[7] Krueger J, Nguyen JT, Dykstra-Aiello CJ, Taishi P (2019). Local sleep. Sleep Med Rev.

[8] Krone LB, Vyazovskiy VV (2020). Unresponsive or just asleep? Do local slow waves in the perilesional cortex have a function?. Brain.

[9] Beltramo R (2013). Layer-specific excitatory circuits differentially control recurrent network dynamics in the neocortex. Nat Neurosci.

[10] Chauvette S, Volgushev M, Timofeev I (2010). Origin of Active States in Local Neocortical Networks during Slow Sleep Oscillation. Cereb Cortex.

[11] Sanchez-Vives MV, McCormick DA (2000). Cellular and network mechanisms of rhytmic recurrent activity in neocortex. Nat Neurosci.

[12] Buzsáki G (2002). Theta Oscillations in the Hippocampus. Neuron.

[13] Boyce R, Glasgow SD, Williams S, Adamantidis A (2016). Causal evidence for the role of REM sleep theta rhythm in contextual memory consolidation. Science.

[14] Sanchez-Vives, McCormick DA, Sanchez-Vives MV, McCormick DA (2000). Cellular and network mechanisms of rhythmic recurrent activity in neocortex. Nat Neurosci.

[15] Hoerder-Suabedissen A (2019). Cell-Specific Loss of SNAP25 from Cortical Projection Neurons Allows Normal Development but Causes Subsequent Neurodegeneration. Cereb Cortex.

[16] Gerfen CR, Paletzki R, Heintz N (2013). GENSAT BAC cre-recombinase driver lines to study the functional organization of cerebral cortical and basal ganglia circuits. Neuron.

[17] Lefort S, Tomm C, Floyd Sarria J-C, Petersen CCH (2009). The Excitatory Neuronal Network of the C2 Barrel Column in Mouse Primary Somatosensory Cortex. Neuron.

[18] Vecchia D (2020). Temporal Sharpening of Sensory Responses by Layer V in the Mouse Primary Somatosensory Cortex. Curr Biol.

[19] Washbourne P (2002). Genetic ablation of the t-SNARE SNAP-25 distinguishes mechanisms of neuroexocytosis. Nat Neurosci.

[20] Funk CM, Honjoh S, Rodriguez AV, Cirelli C, Tononi G (2016). Local slow waves in superficial layers of primary cortical areas during REM sleep. Curr Biol.

[21] Huber R, Deboer T, Tobler I (2000). Effects of sleep deprivation on sleep and sleep EEG in three mouse strains: empirical data and simulations. Brain Res.

[22] Franken P, Dijk DJ, Tobler I, Borbely AA (1991). Sleep deprivation in rats: effects on EEG power spectra, vigilance states, and cortical temperature. Am J Physiol Integr Comp Physiol.

[23] Huber R, Deboer T, Tobler I (2000). Topography of EEG Dynamics After Sleep Deprivation in Mice. J Neurophysiol.

[24] Albrecht U, Foster RG (2002). Placing ocular mutants into a functional context: a chronobiological approach. Methods.

[25] Frank MG, Heller HC (2019). The Function(s) of Sleep. Handbook of Experimental Pharmacology.

[26] Morairty SR (2013). A role for cortical nNOS/NK1 neurons in coupling homeostatic sleep drive to EEG slow wave activity. Proc Natl Acad Sci U S A.

[27] Tossell K (2020). Sleep deprivation triggers somatostatin neurons in prefrontal cortex to initiate nesting and sleep via the preoptic and lateral hypothalamus. bioRxiv.

[28] Prasad JA, Carroll BJ, Sherman SM (2020). Layer 5 Corticofugal Projections from Diverse Cortical Areas: Variations on a Pattern of Thalamic and Extrathalamic Targets. J Neurosci.

[29] Massimini M, Huber R, Ferrarelli F, Hill S, Tononi G (2004). The sleep slow oscillation as a traveling wave. J Neurosci.

[30] Krone L (2017). Top-down control of arousal and sleep: Fundamentals and clinical implications. Sleep Med Rev.

[31] Gent TC, Bandarabadi M, Herrera CG, Adamantidis A (2018). Thalamic dual control of sleep and wakefulness. Nat Neurosci.

[32] Hayashi Y (2015). Cells of a common developmental origin regulate REM/non-REM sleep and wakefulness in mice. Science.

[33] Thomas CW, Guillaumin MCC, McKillop LE, Achermann P, Vyazovskiy VV (2020). Global sleep homeostasis reflects temporally and spatially integrated local cortical neuronal activity. Elife.

[34] Sheroziya M, Timofeev I (2014). Global intracellular slow-wave dynamics of the thalamocortical system. J Neurosci.

[35] Verhage M (2000). Synaptic Assembly of the Brain in the Absence of Neurotransmitter Secretion. Science.

[36] Molnár Z (2002). Normal Development of Embryonic Thalamocortical Connectivity in the Absence of Evoked Synaptic Activity. J Neurosci.

[37] Krueger J, Obál F (1993). A neuronal group theory of sleep function. J Sleep Res.

[38] McKillop LE (2018). Effects of Aging on Cortical Neural Dynamics and Local Sleep Homeostasis in Mice. J Neurosci.

[39] Sakata S, Harris KD (2009). Laminar Structure of Spontaneous and Sensory-Evoked Population Activity in Auditory Cortex. Neuron.

[40] Brown L, Hasan S, Foster RG, Peirson SN (2016). COMPASS: Continuous Open Mouse Phenotyping of Activity and Sleep Status. Wellcome open Res.

[41] Schmid B, Helfrich-Förster C, Yoshii T (2011). A New ImageJ Plug-in “ActogramJ” for Chronobiological Analyses. J Biol Rhythms.

[42] Howell GT, Lacroix GL (2012). Decomposing interactions using GLM in combination with the COMPARE, LMATRIX and MMATRIX subcommands in SPSS. Tutor Quant Methods Psychol.

[43] Howell DC (2012). Statistical Methods for Psychology.

[44] Achermann P, Borbély A (1998). A. Coherence analysis of the human sleep electroencephalogram. Neuroscience.

[45] Tabachnick B, Fidell L (2011). Multivariate Analysis of Variance (MANOVA). International Encyclopedia of Statistical Science.

[46] Magill PJ (2006). Changes in Functional Connectivity within the Rat Striatopallidal Axis during Global Brain Activation In Vivo. J Neurosci.

[47] Paxinos G, Franklin KBJ (2019). Paxinos and Franklin’s the mouse brain in stereotaxic coordinates.

[48] Schindelin J (2012). Fiji: an open source platform for biological image analysis. Nat Methods.

[49] Grant E, Hoerder-Suabedissen A, Molnar Z (2016). The Regulation of Corticofugal Fiber Targeting by Retinal Inputs. Cereb Cortex.

