# Supplementary material for: A role for the cortex in sleep-wake regulation
Source: Nat Neurosci. Author manuscript; Available in PMC 2021 Dec 19. (PMC7612118; doi:10.1038/s41593-021-00894-6)
Supplement: EMS140496-Supinfo [file EMS140496-supplement-EMS140496_Supinfo.pdf]

A role for the cortex in sleep-wake regulation

- Supplementary Information -

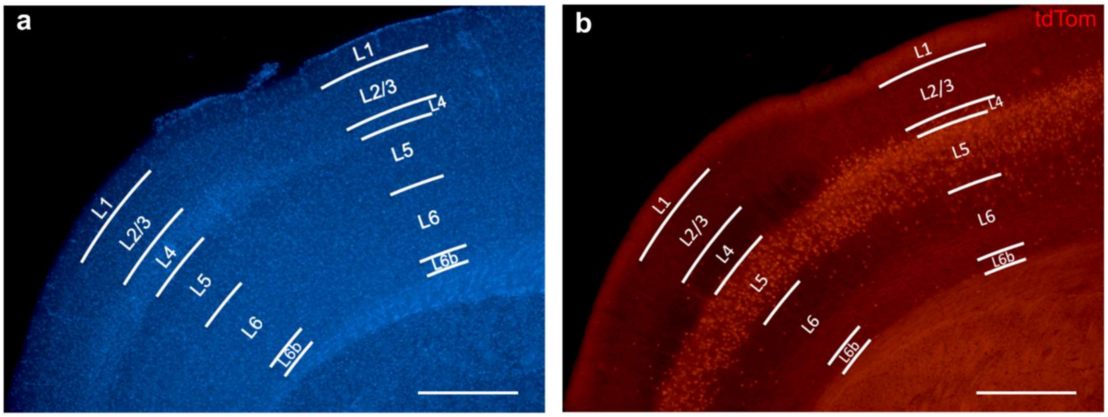

Supplementary Figure 1: Identification of cortical layers in a representative coronal brain slice covering primary motor and sensory cortex.

a) Cortical layers were determined using DAPI staining and identification of characteristic anatomical features of specific layers such as cell density and nuclear size. b) Expression of the red fluorescent protein tdTomato is restricted to layer 5 in both primary motor and sensory cortex. The selective Cre-expression was driven by a Rbp4 promoter that cleaved the STOP-floxed site in the tdTomato reporter Ai14 mouse. Anterior-posterior position: approximately Bregma +0.75 mm.

DAPI: 4′,6-diamidino-2-phenylindole. Scale bars: 500 μm.

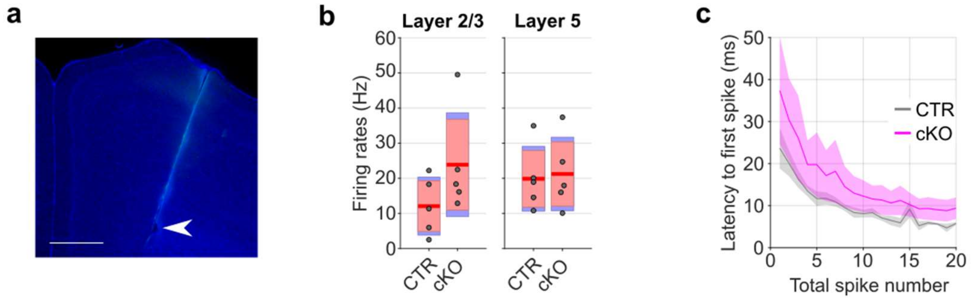

Supplementary Figure 2: Laminar recordings from the transgenic mouse model show no significant genotype differences in laminar firing rates or latency to the first spike in layer 5. a) Insertion tract of a laminar probe on a DAPI counterstained (blue) coronal brain section from a cKO mouse. An electrical microlesion (white arrow), performed under terminal anaesthesia, is visible at the level of the deepest channel of the laminar implant. b) Firing rates across neocortical layers 2/3 and 5 of both genotypes during non-rapid eye movement (NREM) sleep. c) Latency to the first spike at OFF-ON transitions in layer 5 of cKO and CTR animals. No significant genotype differences in the firing rates or in the latency to the first spike at any given spike number were found.

n=5 CTR and n=5 cKO. Data in panel c are presented as mean ± SEM (shaded areas). See Supplementary Table 1 for detailed results.

cKO: conditional knockout animals. CTR: control animals. NREM: Non-rapid eye movement sleep. Scale bar histology image 500µm.

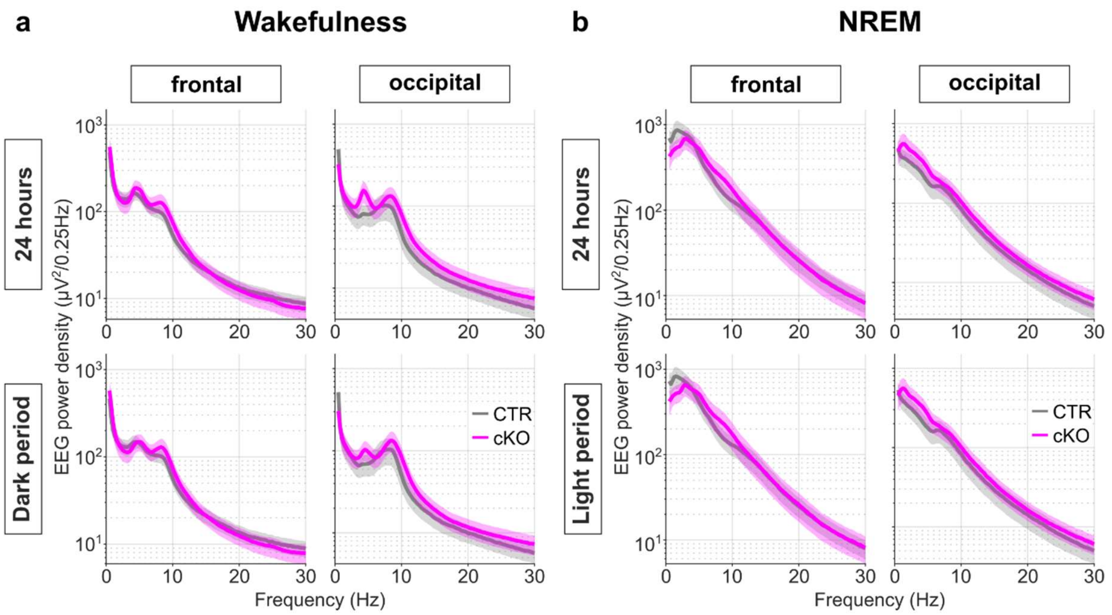

Supplementary Figure 3: EEG power spectra from the frontal and occipital derivations during waking and NREM sleep at baseline show no differences between genotypes.

a) Wake EEG power spectra over 24h and during the dark (active) period. b) NREM EEG power spectra over 24h and during the light (rest) period. No significant genotype differences in either derivation or condition were found.

n=5 CTR and n=8 cKO for EEG spectral analysis. Data in panels a, b are presented as mean ± SEM (shaded areas). See Supplementary Table 1 for detailed results.

cKO: conditional knockout animals. CTR: control animals. EEG: Electroencephalogram. NREM: Non-rapid eye movement sleep.

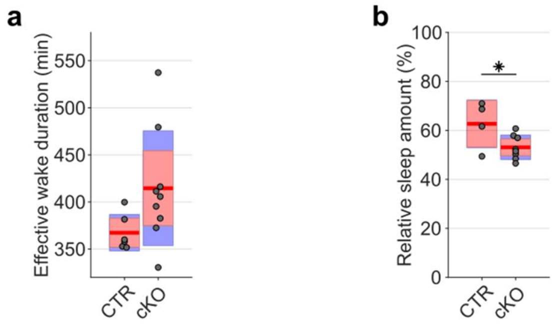

Supplementary Figure 4: Effective wake episode duration and circadian timing of the sleep deprivation experiment influence the amount of rebound sleep.

a) Effective wake duration (wake time during sleep deprivation plus wake bout duration preceding sleep deprivation) in a sleep deprivation experiment performed during the first half of the light period (Zeitgeber time 0-6). b) Relative amount of sleep over 24 hours starting from the beginning of sleep deprivation compared to the preceding 24h interval in a sleep deprivation experiment performed during the second half of the light period (Zeitgeber time 6-12) under passive infrared recordings (PIR).

n=6 CTR and n=9 cKO for sleep deprivation experiment in the EEG setup (panel a), n=4 CTR and n=8 cKO for sleep deprivation experiment in the PIR setup (panel b). Data is presented as group mean (red line), 95% confidence interval (pink box), and one standard deviation (blue box) with individual data points overlaid. See Supplementary Table 1 for detailed results.

cKO: conditional knockout animals. CTR: control animals. EEG: Electroencephalogram. PIR: passive infrared recordings.

Supplementary Table 1: A Summary of ANOVA Results*

* Significant 2-way interactions were followed up with Bonferroni-adjusted post hoc comparisons with $\alpha_{corrected}$ = 0.05/k, where k represents the number of post hoc comparisons; however, Bonferroni correction was not applied to post hoc comparisons involving EEG spectral frequency bins and $\alpha_{uncorrected}$ = 0.05 was adopted. For these spectral analyses we report frequency bins with significant differences in post-hoc comparison before ($\alpha_{uncorrected}$) and after Bonferroni adjustment of α ($\alpha_{corrected}$). All post hoc comparisons were conducted as 1-way ANOVAs involving 2 factor levels; in these cases, the F statistics is equivalent and can be converted to the t statistics by t = √F

Figure	Type of factorial ANOVA; number of post hoc comparisons (*k*)	Results
Fig. 1g	Genotype (CTR vs. cKO) × Time (30 1-ms bins) ANOVA followed by 3 Bonferroni-adjusted post hoc comparisons of Genotype in 10-ms bins	• Genotype × Time interaction $F(29,232)$ = 4.326, $p < 0.001$, with CTR > cKO in the first 10 ms, $p = 0.009$
	1-way Genotype (CTR vs. cKO) ANOVA on time to peak	• Longer surge time in cKO animals $F(1,8)$ = 10.081, $p = 0.013$
Fig. 1h	Genotype (CTR vs. cKO) × Cortical Layer (L2/3 vs. L5) ANOVA on slow-wave amplitude followed by 4 Bonferroni-adjusted post hoc comparisons, including simple effects of Genotype in L2/3 and L5 and simple effects of Cortical Layer in CTR and cKO	• Genotype × Cortical Layer interaction $F(1,8)$ = 95.172, $p < 0.001$. Post hoc comparisons by layer:<br>○ CTR > cKO in L5, $p = 0.006$<br>○ cKO > CTR in L2/3, $p = 0.058$<br>Post hoc comparisons by genotype:<br>○ L5 > L2/3 in CTR, $p < 0.001$<br>○ L5 > L2/3 in cKO, $p = 0.030$
	1-way Genotype (CTR vs. cKO) ANOVA on L5 to L2/3 slow wave amplitude ratio	• Main effect of Genotype on slow wave amplitude ratio $F(1,8)$ = 10.835, $p = 0.011$
Fig. 1i	Genotype (CTR vs. cKO) × Cortical Layer (L2/3 vs. L5) ANOVA on laminar slow-wave activity followed by 4 Bonferroni-adjusted post hoc comparisons, including simple effects of Genotype in L2/3 and L5 and simple effects of Cortical Layer in CTR and cKO	• Genotype × Cortical Layer interaction $F(1,8)$ = 114.820, $p < 0.001$<br>Post hoc comparisons by layer:<br>○ CTR > cKO in L5, $p = 0.021$<br>○ cKO > CTR in L2/3, $p = 0.015$<br>Post hoc comparisons by genotype:<br>○ L5 > L2/3 in CTR, $p < 0.001$<br>○ L5 > L2/3 in cKO, $p = 0.385$
	1-way Genotype (CTR vs. cKO) ANOVA on L5 to L2/3 slow wave activity ratio	• Main effect of Genotype on slow wave activity ratio $F(1,8)$ = 53.68, $p < 0.001$
	Genotype (CTR vs. cKO) x Cortical Layer (L2/3 vs. L5) × Spectral Frequency (119 0.25-Hz bins) ANOVAs on LFP spectra NREM sleep followed by Cortical Layer x Spectral Frequency ANOVAs for each genotype separately and uncorrected post hoc comparisons of Layers in 119 0.25-Hz bins	• Genotype x Cortical Layer x Spectral Frequency interaction $F(118,944)$ = 24.842, $p < 0.001$<br>• Cortical Layer × Spectral Frequency interaction, $F(118,472)$ = 87.078, $p < 0.001$, L5 > L2/3 in frequency bins 1-32, ps < 0.05 ($\alpha_{uncorrected}$), frequency bins 4-13 ($\alpha_{corrected}$), bin size 0.25 Hz with bin 1=0.5 Hz and bin 119=30 Hz<br>• Cortical Layer × Spectral Frequency interaction, $F(118,472)$ = 11.753, $p < 0.001$, L5 > L2/3 in frequency bins 6, 7, L2/3 > L5

		in frequency bins 14-119, *p*s < 0.05 ($α_{uncorrected}$), frequency bins 19-24 ($α_{corrected}$), bin size 0.25 Hz with bin 1=0.5 Hz and bin 119=30 Hz
Fig. 1j	Genotype (CTR vs. cKO) × Spectral Frequency (119 0.25-Hz bins) ANOVAs on frontal EEG spectra during wakefulness<br><br>Genotype (CTR vs. cKO) × Spectral Frequency (119 0.25-Hz bins) ANOVAs on frontal EEG spectra during NREM sleep	• Genotype × Spectral Frequency interaction $F(118,1298) = 1.998$, $p < 0.001$, but no significant effect of Genotype in any of the 119 0.25-Hz bins, *p*s > 0.5<br>• Genotype × Spectral Frequency interaction $F(118,1298) = 2.793$, $p < 0.001$, but no significant effect of Genotype in any of the 119 0.25-Hz bins, *p*s > 0.2
Fig. 2c	Genotype (CTR vs. cKO) × Vigilance State (Wake, NREM, and REM) ANOVA followed by 3 Bonferroni-adjusted post hoc comparisons of Genotype under the three vigilance states	• Genotype × Vigilance State interaction Greenhouse-Geisser $F(1.056,13.732) = 33.008$, $p < 0.001$, with cKO > CTR in Wake state, $p < 0.001$, and CTR > cKO in NREM state, $p < 0.001$
Fig. 2d	Genotype (CTR vs. cKO) × Time (12 2-h bins) ANOVA followed by 6 Bonferroni-adjusted post hoc comparisons of Genotype in 4-h bins	• Main effect of Genotype $F(1,13) = 30.804$, $p < 0.001$ and Genotype × Time interaction Greenhouse-Geisser $F(5,67) = 3.467$, $p = 0.007$, with cKO > CTR for the entire 12-h night, *p*s < 0.005, cKO > CTR also approaching significance ($α_{corrected}$: $p = 0.008$) from ZT0 h to ZT4 h, $p = 0.023$
Fig. 2e	Separate 1-way Genotype (CTR vs. cKO) ANOVAs on maximum and average wake duration	• Main effect of Genotype on maximum wake duration $F(1,13) = 11.326$, $p = 0.005$ and main effect of Genotype on average wake duration $F(1,13) = 24.392$, $p < 0.001$
Fig. 2f	General linear model with 5 factors: MouseID, MouseID x Episode Duration (random factors), Genotype, Episode Duration, and Genotype x Episode Duration (fixed factors); dependent variable: SWA Ratio post/pre sleep;	• Main effect of Genotype on SWA Ratio post/pre sleep $F(1,57.37) = 6.44$, $p = 0.014$
Fig. 3b	Genotype (CTR vs. cKO) × Time (12 30-min bins) ANOVA followed by 3 Bonferroni-adjusted post hoc comparisons of Genotype in 2-h bins	• Genotype × Time interaction $F(11,121) = 7.561$, $p < 0.001$, with CTR > cKO in the first 2 h, $p < 0.001$
Fig. 3c	Genotype (CTR vs. cKO) × Spectral Frequency (119 0.25-Hz bins) ANOVA followed by uncorrected post hoc comparisons of Genotype in 119 0.25-Hz bins	• Genotype × Spectral Frequency interaction $F(118,1298) = 4.068$, $p < 0.001$, due to CTR > cKO in frequency bins 2–14, 17–19, 32–36, 38, 42, 99–102, 107, and 110, *p*s < 0.05 ($α_{uncorrected}$), frequency bins 4-6, 9, 10 ($α_{corrected}$), bin size 0.25 Hz with bin 1=0.5 Hz and bin 119=30 Hz
Figs. 3e and 3f	Genotype (CTR vs. cKO) multivariate ANOVA with 3 response variables, including period length (τ), periodogram power (Qp), and phase shift ($\Delta\varphi$)	• No main effect of Genotype, Wilks' λ $F(3,13) = 0.487$, $p = 0.697$; τ: $F(1,15) = 0.695$, $p = 0.418$; Qp: $F(1,15) = 0.304$, $p = 0.590$; and $\Delta\varphi$: $F(1,15) = 0.399$, $p = 0.537$

Ext. Data Fig. 1a	Cortical Layer (L2/3 vs. L5) × Time (30 1-ms bins) ANOVA followed by 3 Bonferroni-adjusted post hoc comparisons of Cortical Layer in 10-ms bins	• Cortical Layer × Time interaction $F(29,174) = 9.412$, $p < 0.001$, with L5 > L2/3 in the first 10 ms, $p = 0.008$
Ext. Data Fig. 1b	Cortical Layer (L2/3 vs. L5) × Count (20 1-spike bins) ANOVA followed by 10 Bonferroni-adjusted post hoc comparisons of Cortical Layer in 2-spike bins	• Main effect of Cortical Layer $F(1,6)$ 86.301, $p < 0.001$ and Cortical Layer × Count interaction $F(17,102) = 27.205$, $p < 0.001$, with L5 < L2/3 in all spike count bins, ps < 0.001
Suppl. Fig. 2b	Genotype (CTR vs. cKO) × Layer (L2/3 vs. L5) ANOVA followed by 2 Bonferroni-adjusted post hoc comparisons of Genotype for the two layers	• No Genotype × Layer interaction $F(1,8) = 0.946$, $p = 0.359$, no significant effect of Genotype in any of the layers, ps > 0.1
Suppl. Fig. 2c	Genotype (CTR vs. cKO) × Count (16 1-spike bins) ANOVA followed by 16 Bonferroni-adjusted post hoc comparisons of Genotype. Note: spike counts 17-20 were excluded from the ANOVA due to insufficient data in one CTR animal	• No Genotype × Count interaction $F(15,120) = 0.750$, $p = 0.730$, no significant effect of Genotype in any of the spike count bins, ps > 0.1
Suppl. Fig. 3a	Genotype (CTR vs. cKO) × Spectral Frequency (119 0.25-Hz bins) ANOVAs on frontal and occipital wake EEG spectra during baseline	• For frontal and occipital EEG spectra during the 24-h period, Genotype × Spectral Frequency interactions $F(118,1298) = 1.998$, $p < 0.001$ and $F(118,1298) = 2.730$, $p < 0.001$, but no significant effect of Genotype in any of the 119 0.25-Hz bins, ps > 0.05<br>• For frontal and occipital EEG spectra during the dark period, Genotype × Spectral Frequency interactions $F(118,1298) = 1.614$, $p < 0.001$ and $F(118,1298) = 1.905$, $p < 0.001$, but no significant effect of Genotype in any of the 119 0.25-Hz bins, ps > 0.05
Suppl. Fig. 3b	Genotype (CTR vs. cKO) × Spectral Frequency (119 0.25-Hz bins) ANOVAs on frontal and occipital NREM EEG spectra during baseline	• For frontal EEG spectra during the 24-h period, Genotype × Spectral Frequency interaction $F(118,1298) = 2.793$, $p < 0.001$, but no significant simple effect of Genotype in any of the 119 0.25-Hz bins, ps > 0.05<br>• For occipital EEG spectra during the 24-h period, no significant main effect of Genotype or Genotype × Spectral Frequency interaction, $p > 0.7$<br>• For frontal EEG spectra during the light period, Genotype × Spectral Frequency interaction $F(118,1298) = 2.693$, $p < 0.001$, but no significant simple effect of Genotype in any of the 119 0.25-Hz bins, ps > 0.05<br>• For occipital EEG spectra during the light period, no significant main effect of Genotype or Genotype × Spectral Frequency interaction $F(118,1298) = 1.193$, $p = 0.086$, no significant simple effect of

		Genotype in any of the 119 0.25-Hz bins, *p*s > 0.05
Ext. Data Fig. 5	Genotype (CTR vs. cKO) × EEG Derivation (Frontal vs. Occipital) ANOVA on peak theta frequency followed by 2 post hoc comparisons of Genotype for frontal and occipital EEG	• Main effect of Genotype $F(1,11) = 33.532$, $p < 0.001$ and Genotype × EEG Derivation interaction $F(1,11) = 14.233$, $p = 0.003$<br>• CTR > cKO for both frontal and occipital EEG-derived peak theta frequency, $p < 0.001$ and $p = 0.01$, respectively
Ext. Data Fig. 6	Genotype (CTR vs. cKO) × Phase (Light vs. Dark) × Vigilance State (Wake, NREM, and REM) ANOVA, followed by Genotype (CTR vs. cKO) × Phase (Day vs. Night) ANOVAs for each vigilance state and 6 Bonferroni-adjusted post hoc comparisons of Genotype	• 3-way Genotype × Phase × Vigilance State interaction Greenhouse-Geisser $F(1,14) = 36.083$, $p < 0.001$<br>• For Wake state, Genotype × Phase interaction $F(1,13) = 36.961$, $p < 0.001$, with cKO > CTR at night, $p < 0.001$<br>• For NREM state, Genotype × Phase interaction $F(1,13) = 36.352$, $p < 0.001$, with CTR > cKO at night, $p < 0.001$, CTR > cKO approaching significance at day ($\alpha_{corrected}$: $p = 0.008$), $p = 0.049$<br>• For REM state, Genotype × Phase interaction approached significance, $F(1,13) = 3.703$, $p = 0.076$, with CTR > cKO at night, $p = 0.001$
Suppl. Fig. 4a	1-way Genotype (CTR vs. cKO) ANOVA on effective wake duration	• Main effect of Genotype on effective wake duration approached significance, $F(1,13) = 3.325$, $p = 0.091$
Suppl. Fig. 4b	1-way Genotype (CTR vs. cKO) ANOVA on relative sleep amount	• Main effect of Genotype on relative sleep amount , $F(1,10) = 5.405$, $p = 0.042$
Ext. Data Fig. 7a	Genotype (CTR vs. cKO) × Vigilance State (Wake, NREM, and REM) ANOVA followed by 3 Bonferroni-adjusted post hoc comparisons of Genotype under the three vigilance states	• Genotype × Vigilance State interaction Greenhouse-Geisser $F(1,14) = 27.754$, $p < 0.001$, with cKO > CTR in Wake state, $p < 0.001$, CTR > cKO in NREM state, $p < 0.001$, and CTR > cKO in REM state, $p = 0.015$
Ext. Data Fig. 7b	Genotype (CTR vs. cKO) × Time (12 2-h bins) ANOVA followed by 4 Bonferroni-adjusted post hoc comparisons of Genotype in 6-h bins	• Main effect of Genotype $F(1,13) = 25.540$, $p < 0.001$ and Genotype × Time interaction Greenhouse-Geisser $F(4,54) = 4.222$, $p = 0.004$, with cKO > CTR for the entire 12-h night, *p*s <= 0.001
Ext. Data Fig. 7c	Genotype (CTR vs. cKO) × Phase (Light vs. Dark) ANOVA on NREM rebound after sleep deprivation	• No significant effects. No Genotype x Phase interaction $F(1,13) = 0.253$, $p = 0.623$. No effect of Genotype $F(1,13) = 0.657$, $p = 0.432$.
Ext. Data Fig. 7d	1-way Genotype (CTR vs. cKO) ANOVA on change in NREM episode duration	• No significant effect, $F(1,13) = 0.002$, $p = 0.967$
Ext. Data Fig. 8a	Genotype (CTR vs. cKO) × Cortical Layer (L2/3 vs. L5) × Time (12 30-min bins) ANOVA, followed by Genotype (CTR vs. cKO) × Time (3 2-h bins) ANOVAs for L2/3 and L5 and 6 Bonferroni-	• 3-way Genotype × Cortical Layer × Time interaction approached significance, $F(11,88) = 1.803$, $p = 0.065$

	adjusted post hoc comparisons of Genotype in 2-h bins	• For L2/3, Genotype × Time interaction *F*(2,16) = 8.699, *p* = 0.003, with CTR > cKO in first 2 h, *p* = 0.006<br>• For L5, Genotype × Time interaction *F*(2,16) = 9.022, *p* = 0.002, with CTR > cKO in first 2 h, *p* < 0.001
Ext. Data Fig. 8b	Genotype (CTR vs. cKO) × EEG Derivation (Frontal vs. Occipital) × Time (12 30-min bins) ANOVA, followed by Genotype (CTR vs. cKO) × Time (3 2-h bins) ANOVAs for frontal and occipital EEG and 6 Bonferroni-adjusted post hoc comparisons of Genotype in 2-h bins	• 3-way Genotype × EEG Derivation × Time interaction approached significance, Greenhouse-Geisser *F*(1.710,18.805) = 3.262, *p* = 0.067<br>• For frontal EEG, Genotype × Time interaction *F*(2,22) = 20.030, *p* < 0.001, with CTR > cKO in first 2 h, *p* < 0.001<br>For occipital EEG, no Genotype × Time interaction Greenhouse-Geisser *F*(1.234,13.574) = 0.078, *p* = 0.835, no significant effect involving Genotype, *p*s > 0.4
Ext. Data Fig 9	Genotype (CTR vs. cKO) × Spectral Frequency (119 0.25-Hz bins) ANOVAs on frontal and occipital wake EEG spectra during sleep deprivation, followed by uncorrected post hoc comparisons of Genotype in 119 0.25-Hz bins	• For frontal wake EEG spectrum, Genotype × Spectral Frequency interaction *F*(118,1298) = 5.807, *p* < 0.001, due to CTR > cKO in frequency bins 31–42, *p*s < 0.05 ($\alpha_{uncorrected}$), frequency bins 36, 37 ($\alpha_{corrected}$), bin size 0.25 Hz with bin 1=0.5 Hz and bin 119=30 Hz<br>• For occipital wake EEG spectrum, Genotype × Spectral Frequency interaction *F*(118,1298) = 10.007, *p* < 0.001, due to CTR > cKO in frequency bins 31–55, 67, 75, 78–81, 83–84, 88, 90, 92–96, and 98–119, *p*s < 0.05 ($\alpha_{uncorrected}$), frequency bins 34-41 ($\alpha_{corrected}$), bin size 0.25 Hz with bin 1=0.5 Hz and bin 119=30 Hz
Ext. Data Fig. 10a	Genotype (CTR vs. cKO) × Lighting (LD vs. DD) ANOVA, followed by 2 Bonferroni-adjusted post hoc comparisons of Genotype under the two Lightning conditions	• Main effect of Genotype *F*(1,15) = 18.604, *p* = 0.001, no interaction Lightning x Genotype *F*(1,15) = 0.116, *p* = 0.738, cKO > CTR in LD condition *p* < 0.001 and DD condition *p* = 0.003
Ext. Data Fig. 10b	Genotype (CTR vs. cKO) × Lighting (LD vs. DD) × Time (12 2-h bins) ANOVA, followed by Genotype (CTR vs. cKO) × Time (6 4-h bins) ANOVAs for each lighting condition and 12 Bonferroni-adjusted post hoc comparisons of Genotype in 4-h bins	• Genotype × Time interaction Greenhouse-Geisser *F*(3.801,57.016) = 3.319, *p* = 0.018<br>• in LD condition cKO > CTR from ZT16h to ZT24 (*p*s < 0.01) and approaching significance from ZT0 h to ZT4 h (*p* = 0.072) and ZT4 h to ZT8 h (*p* = 0.011)<br>• in DD condition cKO > CTR from ZT16h to ZT24 (*p*s < 0.01) and approaching significance from ZT0 h to ZT4 h (*p* = 0.012)
