# Supplementary material for: A role for the cortex in sleep-wake regulation
Source: Nat Neurosci. Author manuscript; Available in PMC 2021 Dec 19. (PMC7612118; doi:10.1038/s41593-021-00894-6)
Supplement: reportingsummary [file EMS140496-supplement-reportingsummary.pdf]

nature research

Corresponding author(s): Vladyslav Vyazovskiy and Zoltan Molnar

Last updated by author(s): 12 June 2021

# Reporting Summary

Nature Research wishes to improve the reproducibility of the work that we publish. This form provides structure for consistency and transparency in reporting. For further information on Nature Research policies, see Authors & Referees and the Editorial Policy Checklist .

## Statistics

For all statistical analyses, confirm that the following items are present in the figure legend, table legend, main text, or Methods section.

| n/a | Confirmed | |
|---|---|---|
| ☐ | ☒ | The exact sample size (*n*) for each experimental group/condition, given as a discrete number and unit of measurement |
| ☐ | ☒ | A statement on whether measurements were taken from distinct samples or whether the same sample was measured repeatedly |
| ☐ | ☒ | The statistical test(s) used AND whether they are one- or two-sided<br>*Only common tests should be described solely by name; describe more complex techniques in the Methods section.* |
| ☒ | ☐ | A description of all covariates tested |
| ☐ | ☒ | A description of any assumptions or corrections, such as tests of normality and adjustment for multiple comparisons |
| ☐ | ☒ | A full description of the statistical parameters including central tendency (e.g. means) or other basic estimates (e.g. regression coefficient) AND variation (e.g. standard deviation) or associated estimates of uncertainty (e.g. confidence intervals) |
| ☐ | ☒ | For null hypothesis testing, the test statistic (e.g. *F*, *t*, *r*) with confidence intervals, effect sizes, degrees of freedom and *P* value noted<br>*Give P values as exact values whenever suitable.* |
| ☒ | ☐ | For Bayesian analysis, information on the choice of priors and Markov chain Monte Carlo settings |
| ☒ | ☐ | For hierarchical and complex designs, identification of the appropriate level for tests and full reporting of outcomes |
| ☐ | ☒ | Estimates of effect sizes (e.g. Cohen's *d*, Pearson's *r*), indicating how they were calculated |

*Our web collection on statistics for biologists contains articles on many of the points above.*

## Software and code

Policy information about availability of computer code

| | |
|---|---|
| Data collection | The software package 'Synapse Suit' (Tucker-Davis Technologies Inc., Alachua, FL, USA) was used for data collection. |
| Data analysis | Custom-made code in Matlab (The MathWorks Inc, Natick, Massachusetts, USA, version v2017a) was used for resampling of rawdata and for further electrophysiological data analysis. Commented Matlab code for key analyses is deposited on Figshare (DOI: 10.6084/m9.figshare.14737578). The software Sleep Sign for Animals (SleepSign Kissei Comtec Co., Ltd., Nagano, Japan, version 3.3.6.1602) was used for sleep scoring and to compute the fast Fourier transform routine. IBM SPSS Statistics for Windows (IBM Corp., Armonk, N.Y., USA, version 25.0) and SAS JMP (SAS Institute Inc. Cary, NC, USA, version 7.0) were used for statistical analysis. ImageJ (Wayne Rasband, National Institutes of Health, USA, version 1.52a) was used to merge fluorescent images and add scale bars. The ImageJ plug-in "ActogramJ" (Benjamin Schmid and Taishi Yoshii, University of Wurzburg, Germany) was used for periodogram analysis. Figures were prepared in Inkscape (Inkscape project, version 1.0.2). |

For manuscripts utilizing custom algorithms or software that are central to the research but not yet described in published literature, software must be made available to editors/reviewers. We strongly encourage code deposition in a community repository (e.g. GitHub). See the Nature Research guidelines for submitting code & software for further information.

## Data

Policy information about availability of data

All manuscripts must include a data availability statement. This statement should provide the following information, where applicable:

- Accession codes, unique identifiers, or web links for publicly available datasets
- A list of figures that have associated raw data
- A description of any restrictions on data availability

Statistical source data for all figures is available for download via the Nature Neuroscience weblink to this paper. Imaging source data is deposited on Figshare (DOI: 10.6084/m9.figshare.14737584). A sample dataset with spectral data and sleep scoring results used to generate key analyses presented in this manuscript is available for download on Figshare (DOI: 10.6084/m9.figshare.14737569). Raw data from electrophysiological and passive infrared recordings are available from the corresponding authors upon reasonable request.

# Field-specific reporting

Please select the one below that is the best fit for your research. If you are not sure, read the appropriate sections before making your selection.

☒ Life sciences ☐ Behavioural & social sciences ☐ Ecological, evolutionary & environmental sciences

For a reference copy of the document with all sections, see nature.com/documents/nr-reporting-summary-flat.pdf

# Life sciences study design

All studies must disclose on these points even when the disclosure is negative.

| | |
|---|---|
| Sample size | Sample sizes were initially based on previous studies (Huber et al., Brain Research, 2000, and Vyazovskiy et al., Nature, 2011). Power calculations were performed after acquisition of pilot data for the Wellcome Trust grant proposal 203971/Z/16/A indicated an effect size of d = 2.75 for the main outcome parameter NREM sleep time over 24 hours. Applying an intended power of 0.9 and an alpha-error probability of 0.01, the power calculations confirmed the initial sample size estimate of n=8 animals per genotype considering an attrition rate of 25%. |
| Data exclusions | No animals or data were excluded. The exact number of animals contributing to specific experiments/analyses is always mentioned in the text and/or figure legends. |
| Replication | The key finding of this study was confirmed in two independent cohorts of animals, using two different recording methods (electroencephalography and passive infrared recordings). |
| Randomization | Animals were retrieved from the breeder colony upon availability. Based on the breeding scheme we expected that male Rbp4-Cre;Ai14;Snap25fl/fl mice only represent on average 1/8 of the animals in each litter. As soon as animals of the desired genotype were available, they were included in the study and the recording capacities were filled with randomly selected Cre-negative controls from the colony, preferably littermates. |
| Blinding | The experimenters were blinded to the genotype of the transgenic animals in all experiments performed with passive infrared recordings. The experimenters were not blinded during the electrophysiological experiments. Sleep scoring could not be performed fully blinded as originally intended because the characteristic features of increased amount of wakefulness and long wake episodes allowed a clear distinction between the two genotypes during the scoring process. |

# Reporting for specific materials, systems and methods

We require information from authors about some types of materials, experimental systems and methods used in many studies. Here, indicate whether each material, system or method listed is relevant to your study. If you are not sure if a list item applies to your research, read the appropriate section before selecting a response.

### Materials & experimental systems

| n/a | Involved in the study | |
|---|---|---|
| ☐ | ☒ | Antibodies |
| ☒ | ☐ | Eukaryotic cell lines |
| ☒ | ☐ | Palaeontology |
| ☐ | ☒ | Animals and other organisms |
| ☒ | ☐ | Human research participants |
| ☒ | ☐ | Clinical data |

### Methods

| n/a | Involved in the study | |
|---|---|---|
| ☒ | ☐ | ChIP-seq |
| ☒ | ☐ | Flow cytometry |
| ☒ | ☐ | MRI-based neuroimaging |

## Antibodies

| | |
|---|---|
| Antibodies used | anti-MCH antibody (1:2000; H-070-47; Phoenix Pharmaceuticals) |

| | |
|---|---|
| | anti-Hcrt antibody (1:500, kind gift from Anthony Van den Pol, Yale University)<br>anti-rabbit-AlexaFluor488 (1:500, Invitrogen A21206, Lot: 1874771) |
| Validation | All antibodies have been used and validated in previous studies.<br>Validation of the anti-MCH antibody is reported in: Hanriot, L. et al. Characterization of the melanin-concentrating hormone neurons activated during paradoxical sleep hypersomnia in rats. J. Comp. Neurol. 505, 147–157 (2007).<br>Validation of the anti-Hcrt antibody is reported in: van den Pol, A. N., et al. Presynaptic and Postsynaptic Actions and Modulation of Neuroendocrine Neurons by a New Hypothalamic Peptide, Hypocretin/Orexin. J. Neurosci. 18, 7962 LP – 7971 (1998). |

## Animals and other organisms

Policy information about studies involving animals; ARRIVE guidelines recommended for reporting animal research

| | |
|---|---|
| Laboratory animals | Electrophysiology experiments:<br>7 wild type C57BL/6 mice (age at baseline recording 125±8 days)<br>12 Rbp4-Cre;Ai14;Snap25fl/fl mice (age at baseline recording 90±5 days) and 8 Cre-negative littermates (age at baseline recording 85±4 days)<br><br>Passive infrared recordings:<br>10 Rbp4-Cre;Ai14;Snap25fl/fl mice (age at baseline recording 84±1 days) and 7 Cre-negative littermates (age at baseline recording 83±1 days).<br><br>Anatomical work:<br>6 Rbp4-Cre;Ai14;Snap25fl/+ mice (age at perfusion data 57 ± 6 days)<br><br>All experiments were conducted in young adult male mice. A 12:12 h light:dark cycle (lights on at 9 am, light levels 120–180 lux) was implemented, temperature maintained at around 22 ± 2°C, and humidity kept around 50 ± 20%. |
| Wild animals | No wild animals were used in this study. |
| Field-collected samples | The study did not involve samples collected from the field. |
| Ethics oversight | All experiments were performed in accordance with the United Kingdom Animal Scientific Procedures Act 1986 under personal and project licences granted by the United Kingdom Home Office. Ethical approval was provided by the Ethical Review Panel at the University of Oxford. |

Note that full information on the approval of the study protocol must also be provided in the manuscript.

This checklist template is licensed under a Creative Commons Attribution 4.0 International License, which permits use, sharing, adaptation, distribution and reproduction in any medium or format, as long as you give appropriate credit to the original author(s) and the source, provide a link to the Creative Commons license, and indicate if changes were made. The images or other third party material in this article are included in the article's Creative Commons license, unless indicated otherwise in a credit line to the material. If material is not included in the article's Creative Commons license and your intended use is not permitted by statutory regulation or exceeds the permitted use, you will need to obtain permission directly from the copyright holder. To view a copy of this license, visit http://creativecommons.org/licenses/by/4.0/
